# Using the AllerSearch Smartphone App to Assess the Association Between Dry Eye and Hay Fever: mHealth-Based Cross-Sectional Study

**DOI:** 10.2196/38481

**Published:** 2023-09-12

**Authors:** Takenori Inomata, Jaemyoung Sung, Masahiro Nakamura, Masao Iwagami, Yasutsugu Akasaki, Kenta Fujio, Masahiro Nakamura, Nobuyuki Ebihara, Takuma Ide, Masashi Nagao, Yuichi Okumura, Ken Nagino, Keiichi Fujimoto, Atsuko Eguchi, Kunihiko Hirosawa, Akie Midorikawa-Inomata, Kaori Muto, Kumiko Fujisawa, Yota Kikuchi, Shuko Nojiri, Akira Murakami

**Affiliations:** 1 Department of Ophthalmology Juntendo University Graduate School of Medicine Tokyo Japan; 2 Department of Digital Medicine Juntendo University Graduate School of Medicine Tokyo Japan; 3 Department of Hospital Administration Juntendo University Graduate School of Medicine Tokyo Japan; 4 Department of Bioengineering Graduate School of Bioengineering, Precision Health The University of Tokyo Tokyo Japan; 5 Department of Health Services Research Faculty of Medicine University of Tsukuba Ibaraki Japan; 6 Department of Otorhinolaryngology, Head and Neck Surgery Juntendo University Faculty of Medicine Tokyo Japan; 7 Department of Ophthalmology Urayasu Hospital Juntendo University Chiba Japan; 8 Medical Technology Innovation Center Juntendo University Tokyo Japan; 9 Department of Orthopedic Surgery Juntendo University Faculty of Medicine Tokyo Japan; 10 Graduate School of Health and Sports Science Juntendo University Tokyo Japan; 11 Department of Public Policy The Institute of Medical Science, Human Genome Center The University of Tokyo Tokyo Japan

**Keywords:** dry eye, hay fever, mobile health, personalized medicine, smartphone, pollinosis, rhinitis, allergic conjunctivitis, nasal symptom score, nonnasal symptom score, Ocular Surface Disease Index, Japanese Allergic Conjunctival Disease Standard Quality of Life Questionnaire, mobile phone

## Abstract

**Background:**

Dry eye (DE) and hay fever (HF) show synergistic exacerbation of each other’s pathology through inflammatory pathways.

**Objective:**

This study aimed to investigate the association between DE and HF comorbidity and the related risk factors.

**Methods:**

A cross-sectional observational study was conducted using crowdsourced multidimensional data from individuals who downloaded the AllerSearch smartphone app in Japan between February 2018 and May 2020. AllerSearch collected the demographics, medical history, lifestyle and residential information, HF status, DE symptoms, and HF-related quality of life. HF symptoms were evaluated using the nasal symptom score (0-15 points) and nonnasal symptom score (0-12 points). HF was defined by the participants’ responses to the questionnaire as HF, non-HF, or unknown. Symptomatic DE was defined as an Ocular Surface Disease Index total score (0-100 points), with a threshold score of 13 points. HF-related quality of life was assessed using the Japanese Allergic Conjunctival Disease Standard Quality of Life Questionnaire (0-68 points). We conducted a multivariable linear regression analysis to examine the association between the severity of DE and HF symptoms. We subsequently conducted a multivariable logistic regression analysis to identify the factors associated with symptomatic DE (vs nonsymptomatic DE) among individuals with HF. Dimension reduction via Uniform Manifold Approximation and Projection stratified the comorbid DE and HF symptoms. The symptom profiles in each cluster were identified using hierarchical heat maps.

**Results:**

This study included 11,284 participants, classified into experiencing HF (9041 participants), non-HF (720 participants), and unknown (1523 participants) groups. The prevalence of symptomatic DE among individuals with HF was 49.99% (4429/9041). Severe DE symptoms were significantly associated with severe HF symptoms: coefficient 1.33 (95% CI 1.10-1.57; *P*<.001) for mild DE, coefficient 2.16 (95% CI 1.84-2.48; *P*<.001) for moderate DE, and coefficient 3.80 (95% CI 3.50-4.11; *P*<.001) for severe DE. The risk factors for comorbid symptomatic DE among individuals with HF were identified as female sex; lower BMI; medicated hypertension; history of hematologic, collagen, heart, liver, respiratory, or atopic disease; tomato allergy; current and previous mental illness; pet ownership; living room and bedrooms furnished with materials other than hardwood, carpet, tatami, and vinyl; discontinuation of contact lens use during the HF season; current contact lens use; smoking habits; and sleep duration of <6 hours per day. Uniform Manifold Approximation and Projection stratified the heterogeneous comorbid DE and HF symptoms into 14 clusters. In the hierarchical heat map, cluster 9 was comorbid with the most severe HF and DE symptoms, and cluster 1 showed severe HF symptoms with minimal DE-related symptoms.

**Conclusions:**

This crowdsourced study suggested a significant association between severe DE and HF symptoms. Detecting DE among individuals with HF could allow effective prevention and interventions through concurrent treatment for ocular surface management along with HF treatment.

## Introduction

### Background

Dry eye (DE) and hay fever (HF) are common immune-related diseases with chronic negative effects on the quality of life (QoL) and work productivity [1-7]. Both diseases ultimately affect the ocular surface, with the latter manifesting as allergic conjunctivitis. Moreover, they synergistically exacerbate the pathology of the other through inflammatory pathways [7,8]. Both diseases have high comorbidity [9], which could be attributed to overlapping environmental risk factors, lifestyles, and behaviors [10]. The symptoms of DE and HF are heterogeneous and tend to overlap [11-14]. The symptoms of both diseases can lead to contact lens (CL) discontinuation [15,16]; therefore, it is important to concomitantly treat DE and HF to prevent ocular damage and QoL decline. However, cross-disciplinary clinical assessment of the comorbidity of DE and HF has not been achieved because of the diversity of symptoms and the involvement of various departments, such as ophthalmology, otorhinolaryngology, dermatology, and allergy [17-19].

Patient-reported outcomes (PROs) are increasingly valuable data sources that can be used to quantify a patient’s health perception for clinical and research use [20-22]. However, PROs often vary depending on the health and treatment experiences, and this phenomenon is known as a response shift [23]. Response shift may interfere with the interpretation of PROs and the consistency between PROs and clinician-reported outcomes (ClinROs) [22]. Therefore, PROs should be evaluated after detecting and considering response shifts. Accurately measured ClinROs can quantify a patient’s health status and relate it with that of others. However, as in the case of DE, some cases show inconsistencies between PROs and ClinROs [24]. The World Health Organization defined health as a state of complete physical, social, and mental well-being and not merely the absence of disease or infirmity [25]. Thus, reviewing the patient’s history, PROs, and ClinROs to elucidate the pathology and severity is crucial [26], and it is important to concurrently assess the PROs and ClinROs [27,28]. DE and HF manifest as ocular itching, redness, and dryness [9]. Moreover, their symptoms vary across patients and severity; therefore, clinicians should evaluate all disease aspects continuously and provide personalized advice and treatment [17,18].

As a digital extension of PROs, there has been increasing traction for electronic PROs (ePROs) used as an element of PRO data regarding symptoms in the field of mobile health (mHealth) [29]. mHealth is well suited for collecting comprehensive individualized data, such as demographics, lifestyle habits, continuous PROs, behavior, and digitalized biosensing data [11-13,30]. The collection of comprehensive individualized data has been further supported by the increased development of mHealth apps, especially for chronic illnesses and allergic diseases [13,17,31]. We developed 2 in-house ePRO-embedded smartphone apps, DryEyeRhythm and AllerSearch, to elucidate the diversity and heterogeneity of diseases such as DE and HF, respectively [11,14,32]. Our previous study using AllerSearch demonstrated that HF, which was previously considered a singular disease with heterogeneous presentations, was found to exhibit 10 stratified clusters with a combination of 9 HF symptoms based on the nasal symptom score (NSS) and non-NSS (NNSS) [13,33,34]. This demonstrates the potential utility of mHealth in elucidating symptom-based subgroups with highly variable diseases. By analyzing comprehensive individual information collected via mHealth, mHealth may allow elucidation of the pathologic link between DE and HF symptoms, which could facilitate the development of more effective prevention and treatment regimens for patients undergoing ClinRO assessments. Previous studies on DE using the DryEyeRhythm smartphone app have demonstrated that HF is a risk factor for DE, further supporting the pathologic link between both diseases [11,12]. However, few studies exist on the comorbidity between DE and HF because collecting both symptoms precisely is challenging owing to their cross-disciplinary characteristics [9,17,35].

### Objectives

This study aimed to assess the association between DE and HF symptom comorbidity as well as the related risk factors using the AllerSearch smartphone app and stratify the heterogeneous presentations with respect to both diseases.

## Methods

### Recruitment Process Using the AllerSearch App

This crowdsourced cross-sectional, observational study involving prospective data collection was conducted using the iOS version of the AllerSearch app between February 1, 2018, and May 1, 2020 [13]. The prospective participants downloaded the AllerSearch app using their own App Store credentials. All the users provided electronic informed consent for participation. No financial compensation was provided for participation in the study. We included participants in Japan who freely downloaded and used the iOS version of AllerSearch and completed the questionnaires. Duplicate users were excluded from the study.

In Japan, the AllerSearch smartphone app was initially developed by the Department of Ophthalmology at the Juntendo University Graduate School of Medicine, Tokyo, Japan. The app is currently owned by the aforementioned department and InnoJin Inc, Tokyo, Japan. The AllerSearch smartphone app was released for iOS on Apple’s App Store on February 1, 2018, and for Android on Google Play on August 26, 2020. The AllerSearch smartphone app is freely available on the App Store and Google Play. AllerSearch is compatible with iOS 13.0 and Android 7.0 and all later versions (as of September 2022). The AllerSearch smartphone app was initially developed in house using ResearchKit, the open-source framework of Apple Inc [13,17], which contains functionalities for ePRO collection with questionnaires in the formats specified by the validated scales. Detailed user data collection information is provided in the following paragraph.

### User Data Collection

**Figure 1:**
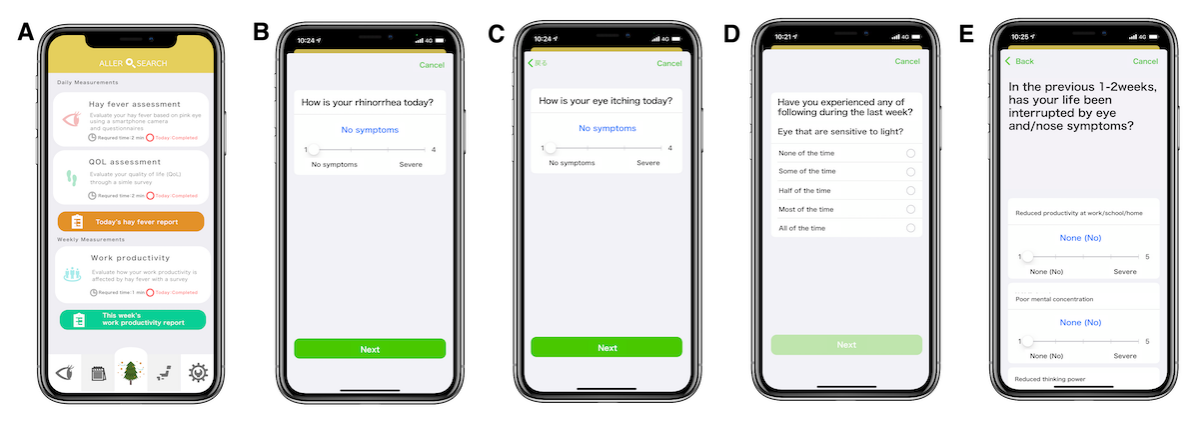
Screenshots of the AllerSearch app. Screenshots of the (A) top screen, (B) nasal symptom score, (C) nonnasal symptom score, (D) Ocular Surface Disease Index, and (E) Japanese Allergic Conjunctival Disease Quality of Life Questionnaire.

The AllerSearch smartphone app collected data regarding the demographic characteristics, medical history, lifestyle and residential information, HF status, preventative behavior for HF (Multimedia Appendix 1), DE symptoms, HF-related QoL, lower eyelid conjunctival hyperemia using the smartphone camera, and location information GPS [32]. The AllerSearch screen is shown in Figure 1A. The participants reported daily disease-specific symptoms, including NSS (Figure 1B) and NNSS (Figure 1C) for HF (Multimedia Appendix 2) [33], the Japanese version of the Ocular Surface Disease Index (J-OSDI) for DE (Figure 1D; Multimedia Appendix 3) [36], and the Japanese Allergic Conjunctival Disease Quality of Life Questionnaire (JACQLQ; Multimedia Appendix 4) for the HF-related QoL score measurement (Figure 1E) [37].

The survey questionnaires in AllerSearch were consensus versions produced by a scientific committee including allergy specialists, ophthalmologists, otolaryngologists, epidemiologists, and the Patient and Public Involvement members [13,17,32,38].

### HF Assessment

The participants were initially classified into the non-HF, HF, and unknown groups based on their responses to the question: “Do you have hay fever?” [13]. HF symptoms were evaluated using NSS and NNSS (Multimedia Appendix 2) [33]. NSS (score range: 0-20) comprised items regarding rhinorrhea, nasal congestion, nasal itching, sneezing, and interference with daily life. NNSS (score range: 0-16) comprised items regarding itchy eyes, watery eyes, eye redness, and itchy ears or mouth. Ocular manifestation was defined as a score of ≥1 in any of the NNSS items 1 to 3. Each NSS and NNSS item was reported on a 4-point Likert scale. The sum of the individual numeric scores for NSS and NNSS was obtained as the total symptom score (TSS; score range: 0-36).

### DE Assessment

Subsequently, all participants were classified into 4 cohorts (normal, mild, moderate, and severe DE). DE symptoms were assessed using the 12-item J-OSDI questionnaire (Multimedia Appendix 3), which evaluates the severity of DE symptoms based on the ocular symptoms, impact on visual functioning, and environmental triggers [36,39]. The J-OSDI total score was based on a 100-point scale, with scores of 0 to 12, 13 to 22, 23 to 32, and 33 to 100 representing normal healthy eyes, mild DE, moderate DE, and severe DE, respectively [40]. Symptomatic DE was defined as a J-OSDI total score of ≥13 [12].

### HF-Related QoL Score Assessment

The QoL score was assessed using JACQLQ [37], which has been validated in Japan and comprises 26 items related to HF symptoms (domain I; 9 items) and QoL (domain II; 17 items; Multimedia Appendix 4). Each item is rated on a 5-point Likert scale. This study used only the 17 items in domain II of JACQLQ for the assessment of HF-related QoL. The total QoL score was calculated as the sum of the 17 QoL-related items (score range: 0-68). The reliability and validity of the app-based and paper-based JACQLQ have been confirmed [34].

### Symptom-Based Stratification for Heterogeneous Symptoms Between DE and HF Comorbidity

DE and HF exhibit various symptoms with synergistic exacerbation of the pathology of the other through inflammatory pathways. Therefore, we stratified the heterogeneous symptoms of DE and HF comorbidity using the NSS, NNSS, and J-OSDI questionnaires. We used the normalized maximum eigengap values to estimate the number of clusters during spectral clustering [41]. We used the Uniform Manifold Approximation and Projection (UMAP) with spectral clustering for dimension reduction based on the 12 items of J-OSDI as well as the 9 items of NSS and NNSS. UMAP was performed using the UMAP-learn Python package (version 0.4.6; Python 3) [42].

### Visualization of Individual DE and HF Symptoms

The hierarchical clustering heat map was used to visualize each surveyed symptom of DE and HF based on the clusters stratified by the dimension reduction algorithm UMAP [14]. The hierarchical clustering heat map, a graphical representation of data with the individual values of NSS, NNSS, and J-OSDI items (0-4) contained in a matrix, was represented as grids of colors with clustering on the rows and columns. The rows were used to group a set of stratified clusters using dimension reduction UMAP. The clustering of the columns was used to observe the correlations between the scores of each NSS, NNSS, and J-OSDI items. A hierarchical clustering heat map was constructed using the *matplotlib* module (version 0.9.0; Python 3).

### Descriptive Statistical Analysis

We assessed the characteristics of patients with nonsymptomatic and symptomatic DE based on the presence of HF (non-HF, HF, and unknown groups) to derive descriptive and inferential statistics. Continuous and categorical variables are presented as median (IQR) and percentages, respectively. The Mann-Whitney *U* test [43] or Kruskal-Wallis test [44] was performed for the continuous variables, whereas the chi-square test [45] was used for the categorical variables. Statistical significance was set at *P*<.05. All data were analyzed using Stata (version 15.1; Stata Corp).

### Inferential Statistical Analysis

Multivariable linear regression analyses [46] were used to assess the association between DE (based on the J-OSDI total score) and HF symptoms (based on TSS), reporting the coefficient and 95% CI. Multivariable logistic regression analyses [47] were conducted to identify the risk factors for symptomatic DE comorbid with HF (vs nonsymptomatic DE comorbid with HF), reporting odds ratio (OR) and 95% CI. The covariates were selected based on a previously established methodology [13,32,38], and all covariates were included in the multivariable linear regression and multivariable logistic analyses. The correlation of the J-OSDI items with the NSS and NNSS items was assessed using the Pearson correlation coefficient [48]. Statistical significance was set at *P*<.05. All data were analyzed using Stata (version 15.1).

### Ethics Approval

This study was approved by the Independent Ethics Committee of the Juntendo University Faculty of Medicine (17-061) and adhered to the tenets of the Declaration of Helsinki.

## Results

### App Downloads and Clinical Study Enrollment

**Figure 2:**
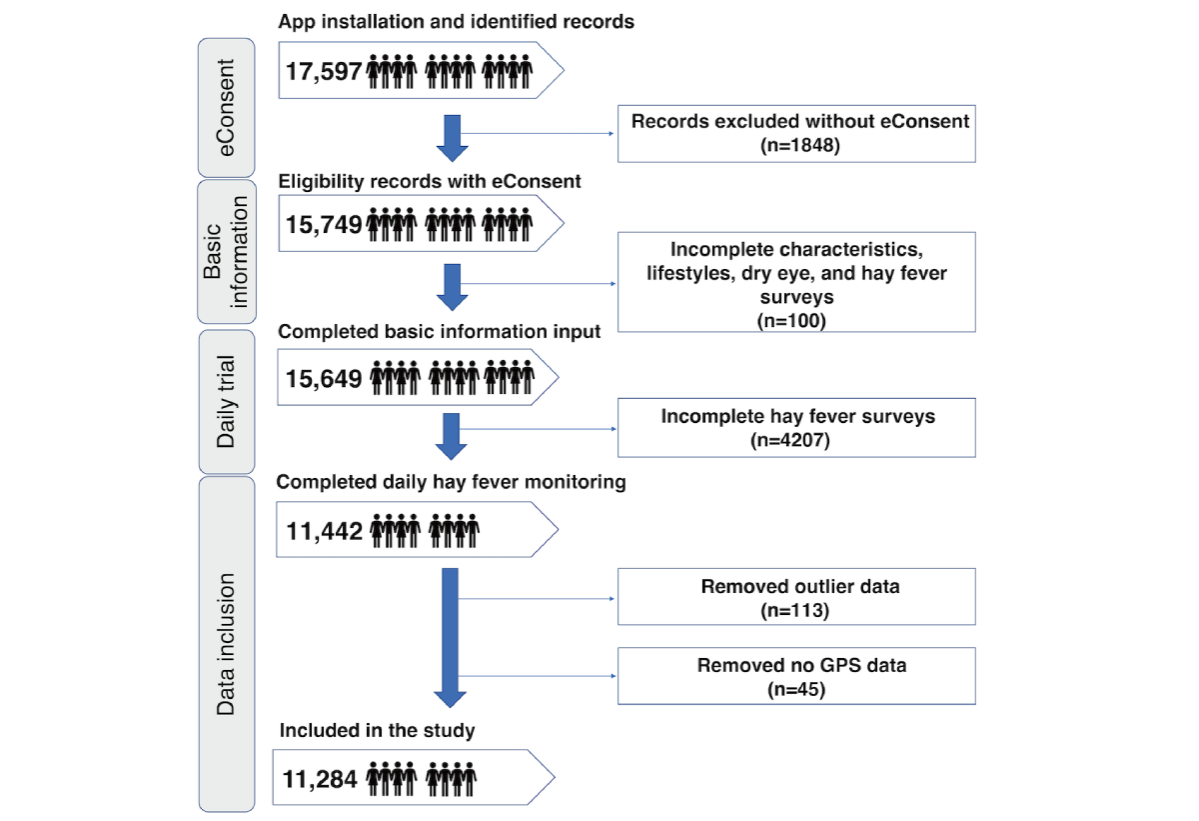
Study cohort description, with the number of participants at each enrollment stage. eConsent: electronic consent.

A flowchart of the study cohort is shown in Figure 2 [13]. We identified 17,597 individuals’ data points between February 1, 2018, and May 1, 2020. Of these 17,597 individuals, 15,749 (89.5%) provided consent, of whom, we excluded 4365 (27.72%) individuals with incomplete daily HF monitoring surveys, outlier data, and without geographic data, leaving 11,284 (64.12%) users.

### Characteristics of the Participants With and Without Symptomatic DE Among Individuals With HF

The characteristics of the participants are provided in Multimedia Appendix 5. Among the individuals with HF, the individuals with symptomatic DE were more likely to be female (*P*<.001) and had a lower BMI (*P*<.001) than the individuals with nonsymptomatic DE. Among the individuals with HF, those treated for hypertension showed a higher prevalence of symptomatic DE than those not treated for hypertension (*P*<.001). Individuals with HF and symptomatic DE had a higher prevalence of diabetes (*P*<.001), hematologic disease (*P*=.003), collagen disease (*P*=.004), liver disease (*P*=.005), respiratory disease (*P*<.001), atopic disease (*P*<.001), tomato allergy (*P*<.001), mental illness (*P*<.001), and pet ownership (*P*<.001) than those without comorbid symptomatic DE. Individuals with HF and symptomatic DE showed a lower prevalence of a history of DE diagnosis than those without comorbid symptomatic DE (*P*<.001). In terms of the associated risk factors related to lifestyle habits, individuals with HF with symptomatic DE had a lower coffee intake (*P*=.03) and were more likely to be current CL users (*P*<.001) and smokers (*P*=.001) as well as have <6 hours of sleep per day (*P*<.001) than those without comorbid symptomatic DE.

Individuals without HF and with symptomatic DE were more likely to be female (*P*<.001) and had a higher prevalence of history of diabetes (*P*=.02), malignant tumor (*P*=.02), mental illness (*P*=.04), history of DE (*P*<.001), and pet ownership (*P*=.006) than those without comorbid symptomatic DE.

### HF Symptoms According to the Severity of DE Symptoms

The prevalence of ocular manifestations was 37.1% (267/720), 64.63% (5843/9041), and 58.77% (895/1523) in the non-HF, HF, and unknown groups, respectively.

Table 1 shows the DE and HF symptoms and QoL among the participants with HF according to the severity of DE symptoms (J-OSDI total score). The median J-OSDI total score for all participants with HF was 12.5 (IQR 6.3-22.9) points. Approximately half (4612/9041, 51.01%) of the participants with HF met the criteria for nonaffected eyes, whereas 24.34% (2201/9041), 11.07% (1001/9041), and 13.57% (1227/9041) of the participants had mild, moderate, and severe DE symptoms, respectively. The median scores regarding indicators of HF symptoms for all participants with HF were 3 (IQR 1-6), 2 (IQR 0-4), and 5 (IQR 2-10) points for the total NSS, total NNSS, and TSS, respectively. The frequency of ocular manifestations (NNSS item 1-3) in individuals with HF assessed using NNSS was positively correlated with the severity of DE symptoms (normal, 2532/4612, 54.9%; mild DE, 1523/2201, 69.2%; moderate DE, 747/1001, 74.63%; and severe DE, 1032/1227, 84.11%). Moreover, there was a positive correlation between the DE and HF symptoms when assessed using TSS and the J-OSDI total scores (TSS: normal, 4 [IQR 1-8]; mild DE, 5 [IQR 2-10]; moderate DE, 7 [IQR 3-12]; and severe DE, 9 [IQR 5-14]; Multimedia Appendix 3).

**Table 1:** Hay fever symptoms and quality of life (QoL) according to the dry eye (DE) severity in the participants with hay fever (n=9041)^a^.

DE symptom status			Normal (n=4612)		Mild DE symptoms (n=2201)		*P* value		Moderate DE symptoms (n=1001)		*P* value		Severe DE symptoms (n=1227)		*P* value		Total
**J-OSDI^b^ (0-100), median (IQR) **																	
	J-OSDI total score	6.3 (2.1-9.1)		18.2 (15.6-20.8)		<.001		27.1 (25-29.2)		<.001		41.7 (37.5-52.1)		<.001		12.5 (6.3-22.9)	
	Ocular symptoms (J-OSDI item 1-5)	5 (0-12.5)		20 (15-25)		<.001		30 (25-35)		<.001		45 (35-55)		<.001		15 (5-25)	
	Vision-related function (J-OSDI item 6-9)	0 (0-0)		8.3 (0-18.8)		<.001		18.8 (6.3-25)		<.001		31.3 (16.7-50)		<.001		6.3 (0-16.7)	
	Environmental triggers (J-OSDI items 10-12)	0 (0-16.7)		25 (16.7-25)		<.001		33.3 (25-50)		<.001		58.3 (41.7-75)		<.001		16.7 (0-25)	
**NSS^c^ (0-4), median (IQR)**																	
	Rhinorrhea (NSS item 1)	1 (0-2)		1 (0-2)		<.001		1 (0-2)		<.001		1 (1-2)		<.001		1 (0-2)	
	Nasal congestion (NSS item 2)	1 (0-1)		1 (0-2)		<.001		1 (0-2)		<.001		1 (0-2)		<.001		1 (0-1)	
	Nasal itching (NSS item 3)	0 (0-1)		1 (0-1)		<.001		1 (0-2)		<.001		1 (0-2)		<.001		1 (0-1)	
	Sneezing (NSS item 4)	1 (0-1)		1 (0-1)		<.001		1 (0-2)		<.001		1 (0-2)		<.001		1 (0-1)	
	Effects on daily life (NSS item 5)	1 (0-1)		1 (0-2)		<.001		1 (0-2)		<.001		1 (1-2)		<.001		1 (0-2)	
	Total NSS (0-20)	3 (1-5)		4 (1-6)		<.001		4 (2-7)		<.001		5 (2-8)		<.001		3 (1-6)	
**NNSS^d^ (0-4)**																	
	Eye itching (NNSS item 1), median (IQR)	0 (0-1)		1 (0-2)		<.001		1 (0-2)		<.001		1 (1-2)		<.001		1 (0-2)	
	Tearing (NNSS item 2), median (IQR)	0 (0-1)		0 (0-1)		<.001		0 (0-1)		<.001		1 (0-2)		<.001		0 (0-1)	
	Eye redness (NNSS item 3), median (IQR)	0 (0-1)		0 (0-1)		<.001		0 (0-1)		<.001		1 (0-1)		<.001		0 (0-1)	
	Ear and mouse itching (NNSS item 4), median (IQR)	0 (0-0)		0 (0-1)		<.001		0 (0-1)		<.001		0 (0-1)		<.001		0 (0-1)	
	Ocular manifestation (yes), n (%)	2532 (54.9)		1523 (69.2)		<.001		747 (74.63)		<.001		1032 (84.11)		<.001		5834 (64.53)	
	Total NNSS (0-16), median (IQR)	1 (0-3)		2 (0-4)		<.001		3 (1-5)		<.001		4 (1-6)		<.001		2 (0-4)	
Total NSS and NNSS (0-36), median (IQR)			4 (1-8)		5 (2-10)		<.001		7 (3-12)		<.001		9 (5-14)		<.001		5 (2-10)
**JACQLQ^e^ QoL score (0-4), median (IQR)**																	
	Reduced productivity at work, school, or home	0 (0-2)		1 (0-2)		<.001		1 (0-2)		<.001		2 (1-3)		<.001		1 (0-2)	
	Poor mental concentration	0 (0-2)		1 (0-2)		<.001		1 (0-2)		<.001		2 (1-3)		<.001		1 (0-2)	
	Reduced thinking power	0 (0-1)		1 (0-2)		<.001		1 (0-2)		<.001		1 (0-3)		<.001		0 (0-2)	
	Impaired reading	0 (0-1)		0 (0-1)		<.001		0 (0-1)		<.001		1 (0-2)		<.001		0 (0-1)	
	Poor memory	0 (0–0)		0 (0-1)		<.001		0 (0-1)		<.001		1 (0-2)		<.001		0 (0-1)	
	Limitation of outdoor life	0 (0-1)		0 (0-2)		<.001		0 (0-2)		<.001		1 (0-3)		<.001		0 (0-2)	
	Limitation of going out	0 (0-1)		0 (0-2)		<.001		1 (0-2)		<.001		1 (0-3)		<.001		0 (0-2)	
	Reluctance to visit friends	0 (0–0)		0 (0-1)		<.001		0 (0-1)		<.001		1 (0-2)		<.001		0 (0-1)	
	Reduced contact with friends	0 (0–0)		0 (0-1)		<.001		0 (0-1)		<.001		0 (0-2)		<.001		0 (0-1)	
	Uneasy with people around you	0 (0–0)		0 (0-1)		<.001		0 (0-1)		<.001		0 (0-2)		<.001		0 (0-1)	
	Impaired sleeping	0 (0-1)		0 (0-2)		<.001		0 (0-2)		<.001		1 (0-3)		<.001		0 (0-2)	
	Tiredness	0 (0-1)		1 (0-2)		<.001		1 (0-2)		<.001		2 (0-3)		<.001		0 (0-2)	
	Fatigue	0 (0-1)		1 (0-2)		<.001		1 (0-3)		<.001		2 (1-3)		<.001		1 (0-2)	
	Frustrated	0 (0-1)		1 (0-2)		<.001		1 (0-2)		<.001		2 (0-3)		<.001		0 (0-2)	
	Irritable	0 (0-1)		0 (0-2)		<.001		1 (0-2)		<.001		1 (0-3)		<.001		0 (0-2)	
	Depressed	0 (0-1)		0 (0-1)		<.001		0 (0-2)		<.001		1 (0-3)		<.001		0 (0-1)	
	Unhappy	0 (0-1)		0 (0-2)		<.001		0 (0-2)		<.001		1 (0-3)		<.001		0 (0-1)	
	QoL total score (0-68)	3 (0-14)		8 (0-21)		<.001		11 (0-26)		<.001		19 (3-36)		<.001		6 (0-20)	

^a^The Kruskal-Wallis test was performed for continuous variables. Statistical significance was set at *P*<.05.

^b^J-OSDI: Japanese version of the Ocular Surface Disease Index.

^c^NSS: nasal symptom score.

^d^NNSS: nonnasal symptom score.

^e^JACQLQ: Japanese Allergic Conjunctival Disease Quality of Life Questionnaire.

### Relationship Between DE and HF Symptoms

Table 2 shows the factors associated with severe HF symptoms based on TSS according to the multivariate linear regression analysis. Severe DE symptoms based on the J-OSDI total score were significantly associated with severe HF symptoms: coefficient 1.33 (95% CI 1.10-1.57; *P*<.001), coefficient 2.16 (95% CI 1.84-2.48; *P*<.001), and coefficient 3.80 (95% CI 3.50-4.11; *P*<.001) for mild, moderate, and severe DE, respectively. In addition, the following covariates were significant (all *P*<.05): younger age, female sex, medicated hypertension, history of liver and respiratory disease, lack of history of systemic disease, mental illness, CL discontinuation during the HF season, lack of current CL use, pet ownership, and the presence of a carpeted floor in the bedroom.

**Table 2:** Association between dry eye and hay fever symptoms according to the multivariate regression analysis (n=11,284)^a^.

Variables				Univariate linear regression				Multivariable linear regression		
				Coefficient (95% CI)		*P* value		Coefficient (95% CI)		*P* value
**J-OSDI^b^ total score**										
	Normal			1 (reference)		N/A^c^		1 (reference)		N/A
	Mild			1.46 (1.21 to 1.70)		<.001		1.33 (1.10 to 1.57)		<.001
	Moderate			2.36 (2.03 to 2.69)		<.001		2.16 (1.84 to 2.48)		<.001
	Severe			4.31 (4.00 to 4.61)		<.001		3.80 (3.50 to 4.11)		<.001
**Demographic characteristics**										
	Age (years), every 1 y			−0.11 (−0.12 to −0.10)		<.001		−0.10 (−0.11 to −0.09)		<.001
	Sex (female vs male)			2.04 (1.84 to 2.25)		<.001		0.96 (0.74 to 1.18)		<.001
	BMI			−0.11 (−0.14 to −0.08)		<.001		0.02 (−0.01 to 0.05)		.13
**Medical history**										
	Medicated hypertension (yes vs no)			−1.80 (−2.25 to −1.35)		<.001		0.62 (0.16 to 1.08)		.008
	**Diabetes**									
		No	1 (reference)		N/A		1 (reference)		N/A	
		Yes	−1.48 (−2.15 to −0.81)		<.001		0.24 (−0.40 to 0.89)		.46	
		Unknown	1.19 (0.83 to 1.56)		<.001		0.35 (0.01 to 0.69)		.045	
	**Systemic disease (yes)**									
		Hematologic disease	−1.11 (−1.97 to −0.25)		.01		0.17 (−0.74 to 1.07)		.72	
		Brain disease	−0.89 (−1.86 to 0.09)		.08		0.54 (−0.47 to 1.55)		.29	
		Collagen disease	0.21 (−1.19 to 1.61)		.77		0.68 (−0.69 to 2.05)		.33	
		Heart disease	−0.87 (−1.59 to −0.16)		.02		0.38 (−0.42 to 1.19)		.35	
		Kidney disease	−0.84 (−1.63 to −0.05)		.04		0.61 (−0.24 to 1.46)		.16	
		Liver disease	−0.10 (−0.93 to 0.72)		.81		1.43 (0.54 to 2.31)		.002	
		Malignant tumor	−1.64 (−2.50 to −0.78)		<.001		0.87 (−0.11 to 1.85)		.08	
		Respiratory disease	0.23 (−0.13 to 0.59)		.20		0.98 (0.30 to 1.66)		.005	
		None	0.56 (0.28 to 0.83)		<.001		1.13 (0.45 to 1.82)		.001	
	Atopic disease			0.75 (0.48 to 1.03)		<.001		0.24 (−0.01 to 0.50)		.06
	Tomato allergy			1.67 (1.02 to 2.33)		<.001		0.59 (−0.02 to 1.20)		.06
	**Mental illness**									
		No	1 (reference)		N/A		1 (reference)		N/A	
		Yes	0.64 (0.18 to 1.09)		.006		−0.46 (−0.89 to −0.03)		.03	
		Previously had	0.41 (−0.03 to 0.85)		.07		−.23 (−0.64 to 0.18)		.27	
**Residential environment**										
	**Living room**									
		Hardwood	1 (reference)		N/A		1 (reference)		N/A	
		Carpet	0.61 (0.35 to 0.87)		<.001		0.07 (−0.20 to 0.34)		.61	
		Tatami (Japanese straw–based floor)	0.49 (0.02 to 0.96)		.04		0.17 (−0.29 to 0.64)		.46	
		Vinyl	−0.49 (−1.23 to 0.26)		.20		−0.57 (−1.45 to 0.31)		.20	
		Other	1.11 (0.20 to 2.02)		.02		0.26 (−0.71 to 1.23)		.60	
	**Bedroom**									
		Hardwood	1 (reference)		N/A		1 (reference)		N/A	
		Carpet	0.60 (0.32 to 0.88)		<.001		0.32 (0.03 to 0.61)		.03	
		Tatami (Japanese straw–based floor)	−0.05 (−0.33 to 0.23)		.71		0.27 (−0.01 to 0.55)		.06	
		Vinyl	−0.37 (−1.23 to 0.47)		.39		−0.35 (−1.35 to 0.65)		.497	
		Other	1.13 (0.25 to 2.00)		.01		0.23 (−0.70 to 1.17)		.62	
	Pet ownership			0.60 (0.38 to 0.82)		<.001		0.26 (0.05 to 0.46)		.01
**Lifestyle habit**										
	Coffee intake (cups/d)			−0.41 (−0.48 to −0.35)		<.001		0.01 (−0.07 to 0.07)		.95
	**Contact lens use**									
		No	1 (reference)		N/A		1 (reference)		N/A	
		Past use	−0.43 (−0.72 to −0.14)		.003		−0.19 (−0.49 to 0.09)		.18	
		Discontinued during the hay fever season	2.52 (1.87 to 3.18)		<.001		1.49 (0.88 to 2.10)		<.001	
		Current use	0.73 (0.49 to 0.96)		<.001		−0.31 (−0.54 to −0.09)		.007	
	Exercise (h/wk)			0.008 (−0.004 to 0.020)		.20		−0.002 (−0.01 to 0.01)		.73
	Bowel movement (times/wk)			−0.03 (−0.05 to −0.001)		.04		−0.0003 (−0.02 to 0.02)		.98
	**Sleep duration (h/d)**									
		<6	0.14 (−0.12 to 0.40)		.28		0.14 (−0.10 to 0.38)		.25	
		6-9	1 (reference)		N/A		1 (reference)		N/A	
		>9	2.57 (1.64 to 3.50)		<.001		0.78 (−0.09 to 1.65)		.08	
	**Smoking habit**									
		No	1 (reference)		N/A		1 (reference)		N/A	
		Yes	−0.08 (−0.37 to 0.21)		.59		0.25 (−0.03 to 0.53)		.08	
		Previously had	−1.08 (−1.38 to −0.79)		<.001		0.22 (−0.06 to 0.51)		.12	
	**Yogurt intake**									
		Rarely	1 (reference)		N/A		1 (reference)		N/A	
		Once/wk	−0.21 (−0.50 to 0.08)		.16		−0.12 (−0.39 to 0.14)		.36	
		Twice or thrice/wk	−0.34 (−0.65 to −0.04)		.03		−0.15 (−0.44 to 0.13)		.29	
		4 or 5 times/wk	−0.71 (−1.1 to −0.31)		<.001		−0.17 (−0.54 to 0.20)		.37	
		Everyday	−1.03 (−1.34 to −0.72)		<.001		−0.03 (−0.33 to 0.27)		.85	

^a^The association between dry eye (based on the Japanese version of the Ocular Surface Disease Index total score) and hay fever symptoms (based on total symptom score) was assessed using univariate and multivariable linear regression analyses. The multivariable linear regression analysis included all covariables.

^b^J-OSDI: Japanese version of the Ocular Surface Disease Index.

^c^N/A: not applicable.

### Risk Factors for HF in Symptomatic DE

Table 3 shows the crude and adjusted ORs of each factor for symptomatic DE in individuals with HF. Compared with nonsymptomatic DE, symptomatic DE showed an independent association with female sex (OR 2.00, 95% CI 1.82-2.20); lower BMI (OR 0.98, 95% CI 0.971-0.996); medicated hypertension (OR 1.29, 95% CI 1.05-1.59); history of atopic disease (OR 1.19, 95% CI 1.06-1.33), hematologic disease (OR 1.82, 95% CI 1.16-2.86), collagen disease (OR 2.36, 95% CI 1.16-4.80), heart disease (OR 1.57, 95% CI 1.04-2.36), liver disease (OR 1.97, 95% CI 1.29-3.00), respiratory disease (OR 1.66, 95% CI 1.17-2.35), current mental illness (OR 2.18, 95% CI 1.78-2.67), previous mental illness (OR 1.44, 95% CI 1.20-1.73), and tomato allergy (OR 1.62, 95% CI 1.23-2.13); discontinuation of CL use during the HF season (OR 1.67, 95% CI 1.29-2.16); current CL use (OR 1.35, 95% CI 1.22-1.50); smoking habits (OR 1.40, 95% CI 1.23-1.59); pet ownership (OR 1.23, 95% CI 1.12-1.35); sleep duration of <6 hours per day (OR 1.29, 95% CI 1.16-1.44); living room furnished with materials other than hardwood, carpet, tatami, and vinyl (OR 0.62, 95% CI 0.39-0.99); and bedroom furnished with materials other than hardwood, carpet, tatami, and vinyl (OR 1.68, 95% CI 1.06-2.67).

**Table 3:** Risk factors for dry eye among individuals with hay fever versus individuals with hay fever without dry eye^a^.

Variables				Crude odds ratio (95% CI)		*P* value		Adjusted odds ratio (95% CI)		*P* value
**Demographic characteristics**										
	Age (years), every 1 y			1.00 (1.00-1.00)		.16		1.00 (1.00-1.01)		.06
	Sex (female vs male)			2.09 (1.92-2.27)		<.001		2.00 (1.82-2.20)		<.001
	BMI			0.98 (0.97-0.99)		.002		0.98 (0.971-0.996)		.01
**Medical history**										
	Medicated hypertension (yes vs no)			1.00 (0.84-1.20)		.96		1.29 (1.05-1.59)		.01
	**Diabetes**									
		No	1 (reference)		N/A^b^		1 (reference)		N/A	
		Yes	0.89 (0.67-1.12)		.41		0.92 (0.68-1.25)		.60	
		Unknown	1.50 (1.29-1.75)		<.001		1.50 (1.28-1.76)		<.001	
	**Systemic disease (yes)**									
		Hematologic disease	1.67 (1.18-2.37)		.004		1.82 (1.16-2.86)		.009	
		Brain disease	1.28 (0.86-1.91)		.23		1.59 (0.97-2.62)		.07	
		Collagen disease	2.47 (1.32-4.61)		.005		2.36 (1.16-4.80)		.02	
		Heart disease	1.32 (0.98-1.78)		.07		1.57 (1.04-2.36)		.03	
		Kidney disease	1.12 (0.82-1.53)		.46		1.25 (0.83-1.91)		.29	
		Liver disease	1.59 (1.15-2.20)		.005		1.97 (1.29-3.00)		.002	
		Malignant tumor	0.90 (0.64-1.28)		.57		0.98 (0.61-1.58)		.94	
		Respiratory disease	1.54 (1.34-1.78)		<.001		1.66 (1.17-2.35)		.005	
		None	0.73 (0.65-0.81)		<.001		1.37 (0.96-1.96)		.09	
	Atopic disease			1.32 (1.19-1.47)		<.001		1.19 (1.06-1.33)		.003
	Tomato allergy			1.96 (0.80-42.8)		.14		1.62 (1.23-2.13)		.001
	**Mental illness**									
		No	1 (reference)		N/A		1 (reference)		N/A	
		Yes	2.57 (2.11-3.13)		<.001		2.18 (1.78-2.67)		<.001	
		Previously had	1.69 (1.41-2.01)		<.001		1.44 (1.20-1.73)		<.001	
**Residential environment**										
	**Living room**									
		Hardwood	1 (reference)		N/A		1 (reference)		N/A	
		Carpet	1.09 (0.98-1.21)		.11		1.08 (0.95-1.22)		.23	
		Tatami (Japanese straw–based floor)	1.15 (0.95-1.38)		.15		1.11 (0.93-1.36)		.33	
		Vinyl	1.51 (1.11-2.07)		.009		1.46 (0.95-2.22)		.08	
		Other	0.77 (0.52-1.14)		.20		0.62 (0.39-0.99)		.046	
	**Bedroom**									
		Hardwood	1 (reference)		N/A		1 (reference)		N/A	
		Carpet	1.04 (0.94-1.17)		.42		0.99 (0.87-1.13)		.89	
		Tatami (Japanese straw–based floor)	1.03 (0.93-1.16)		.55		0.98 (0.86-1.11)		.72	
		Vinyl	1.41 (0.98-2.03)		.06		1.02 (0.62-1.69)		.93	
		Other	1.23 (0.84-1.79)		.29		1.68 (1.06-2.67)		.03	
	Pet ownership			1.35 (1.24-1.47)		<.001		1.23 (1.12-1.35)		<.001
**Lifestyle habits**										
	Coffee intake (cups/d)			0.9 (0.96-1.02)		.42		1.03 (0.99-1.06)		.11
	**Contact lens use**									
		No	1 (reference)		N/A		1 (reference)		N/A	
		Past use	1.21 (1.08-1.36)		.001		1.11 (0.99-1.26)		.07	
		Discontinued during the hay fever season	1.87 (1.46-2.40)		<.001		1.67 (1.29-2.16)		<.001	
		Current use	1.49 (1.35-1.63)		<.001		1.35 (1.22-1.50)		<.001	
	Exercise (h/wk)			1.00 (1.00-1.01)		.09		1.00 (1.00-1.01)		.13
	Bowel movement (times/wk)			0.99 (0.98-1.00)		.16		1.49 (1.00-1.02)		.14
	**Sleep duration (h/d)**									
		<6	1.29 (1.16-1.43)		<.001		1.29 (1.16-1.44)		<.001	
		6-9	1 (reference)		N/A		1 (reference)		N/A	
		>9	1.20 (0.82-1.76)		.34		0.87 (0.59-1.30)		.51	
	**Smoking habit**									
		No	1 (reference)		N/A		1 (reference)		N/A	
		Yes	1.25 (1.11-1.41)		<.001		1.40 (1.23-1.59)		<.001	
		Previously had	0.97 (0.87-1.09)		.64		1.07 (0.94-1.21)		.31	
	**Yogurt intake**									
		Rarely	1 (reference)		N/A		1 (reference)		N/A	
		Once/wk	1.03 (0.92-1.16)		.62		1.00 (0.89-1.13)		.98	
		Twice or thrice/wk	1.07 (0.95-1.20)		.30		1.05 (0.93-1.19)		.44	
		4 or 5 times/wk	1.12 (0.96-1.31)		.16		1.14 (0.97-1.34)		.12	
		Everyday	0.99 (0.88-1.12)		.93		1.04 (0.91-1.18)		.59	

^a^Multivariable logistic regression analyses were conducted to identify the risk factors for symptomatic dry eye (DE) comorbid with hay fever (HF; vs nonsymptomatic DE comorbid with HF), reporting OR and 95% CI. The multivariable logistic analyses included all covariables.

^b^N/A: not applicable.

### Stratification of Individual DE and HF Symptoms

**Figure 3:**
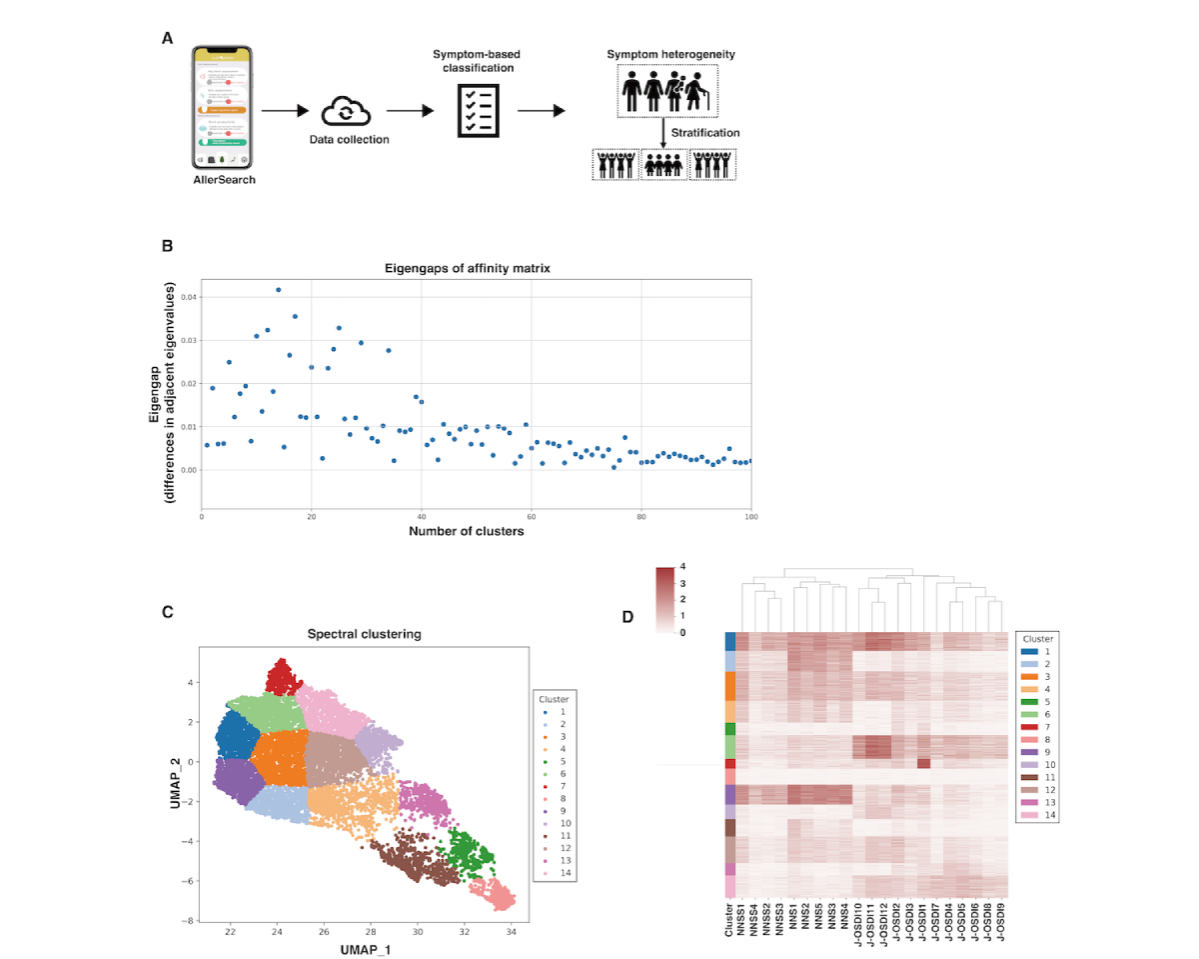
Stratification of the heterogeneous symptoms of dry eye (DE) and hay fever (HF). (A) An overview of the stratification process of heterogeneous and diverse subjective symptoms of DE and HF using AllerSearch. (B) The normalized maximum eigengap values were used to estimate the number of clusters during spectral clustering. Fourteen clusters were determined using the eigengaps of the normalized affinity matrix. (C) Dimension reduction of the included individuals—via Uniform Manifold Approximation and Projection (UMAP) with spectral clustering identified through unsupervised clustering analysis (n=11,284 individuals collected by AllerSearch)—depicted 14 clusters upon stratification for subjective symptoms based on the 12 Japanese version of the Ocular Surface Disease Index (J-OSDI) items and the 9 nasal symptom score (NSS) and non-NSS (NNSS) items. (D) The fraction of individuals within each cluster is visualized on the left-most panel, along with a corresponding heat map of the subjective symptoms of DE and HF in individuals within the identified clusters. The dendrogram clusters for each J-OSDI, NSS, and NNSS item are shown at the top of the heat map.

We conducted a symptom-based stratification of individual DE and HF symptoms. Figure 3A illustrates an overview of the symptom-based stratification process for DE and HF symptoms using AllerSearch [13,14,16]. Figure 3B shows the normalized maximum eigengap values with the estimated number of clusters during spectral clustering [41]; 14 clusters were identified. Figure 3C depicts a UMAP plot [42] of these 14 symptom-based stratified clusters obtained using dimensional reduction analysis. Figure 3D shows a hierarchical heat map [14], demonstrating the individual profiles of DE and HF symptoms based on the stratified clusters.

### Symptoms and QoLs of Each Stratified Cluster

**Figure 4:**
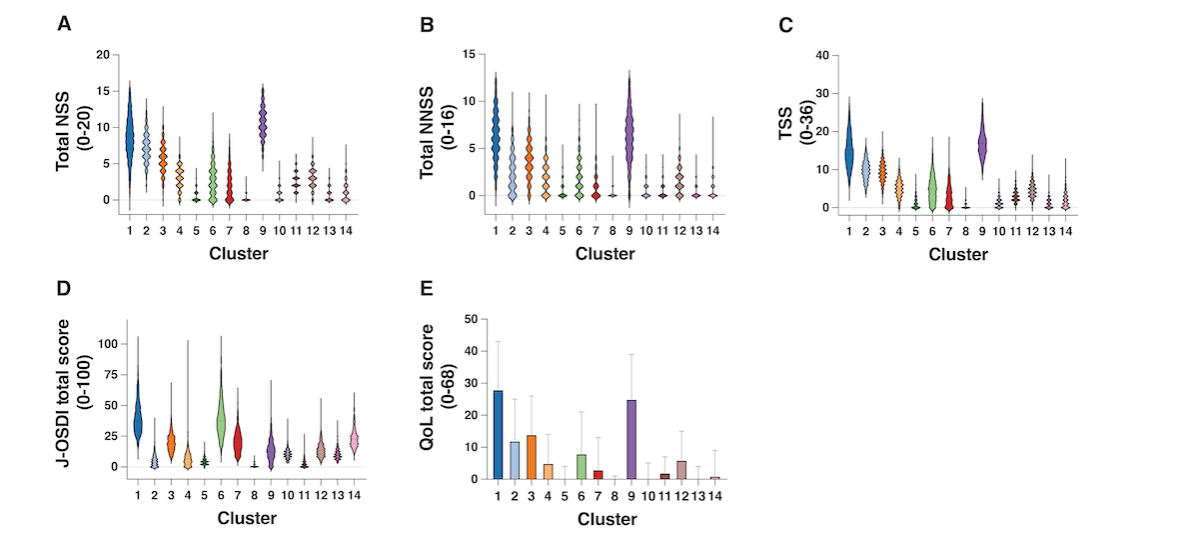
Symptoms and quality of life (QoL) of dry eye and hay fever in each stratified cluster. A bar graph of the (A) total nasal symptom score (NSS), (B) total non-NSS (NNSS), (C) total symptom score (TSS), (D) Japanese version of the Ocular Surface Disease Index (J-OSDI) total score, and (E) QoL total score in each stratified cluster.

Figure 4 shows the total NSS (Figure 4A), total NNSS (Figure 4B), TSS (Figure 4C), J-OSDI total scores (Figure 4D), and QoL total scores (Figure 4E) for each stratified cluster (Multimedia Appendix 6). Cluster 9 showed the most severe HF symptoms (TSS), followed by clusters 1 and 2. Cluster 1 showed the most severe DE symptoms (J-OSDI total score), followed by clusters 6 and 14. Moreover, cluster 1 had the worst QoL total score, followed by clusters 9 and 12.

### Correlation Between Each DE and HF Symptom

**Figure 5:**
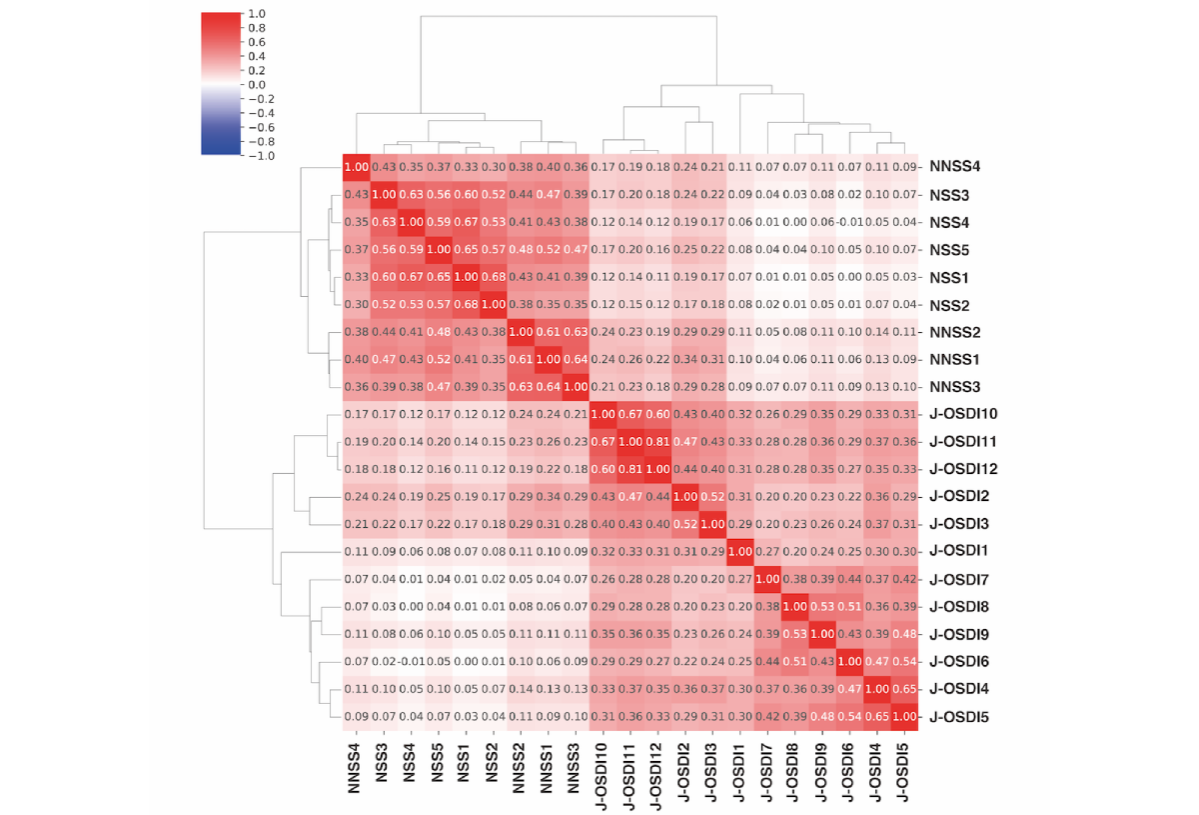
The correlation between each item of Japanese version of the Ocular Surface Disease Index (J-OSDI) and nasal symptom score (NSS) and non-NSS (NNSS). Pearson correlation coefficients between the 12 J-OSDI items and the 9 NSS and NNSS items are shown in the heat map as a color gradient.

Figure 5 shows the correlation heat map between DE and HF symptoms based on 12, 5, and 4 items of the J-OSDI, NSS, and NNSS, respectively. All symptoms were positively correlated with DE and HF; J-OSDI items 2 and 3 showed a stronger association with NNSS items 1, 2, and 3.

## Discussion

### Principal Findings

DE and HF are both ocular inflammatory diseases; moreover, their comorbidity may synergistically worsen the pathology and prognosis. This study identified the high comorbidity between DE and HF using the AllerSearch app and found that DE exacerbation coincides with the worsening of HF symptoms. We identified the specific factors for comorbidity and performed symptom-based stratification to facilitate the treatment of comorbid DE and HF. This study demonstrated the stratification of the variability and heterogeneity of the symptoms among comorbid DE and HF. Our findings may aid prospective individualized and precision medicine for the treatment of various comorbid allergic inflammatory diseases.

### Comparison With Prior Work

Among individuals with HF, the comorbidity rate for symptomatic DE was 48.99% (4429/9041), which was consistent with our previous reports of 52.3% and 52.4% [12,14]. The reported prevalence of DE in patients with allergic conjunctivitis is 0.9% to 97.5% [49-53]. HF is a systemic multiorgan disease resulting from inappropriate immune responses that may present as allergic conjunctivitis, rhinitis, and dermatitis. Therefore, the developmental course of allergic conditions, including dermatitis, food allergy, asthma, and rhinitis, requires monitoring from infancy to adulthood [54]. There are no comprehensive data regarding the epidemiology of DE and HF [17,18]. Accordingly, this is among the first studies to holistically analyze big data regarding HF. Nearly half of the individuals with HF may present with symptomatic DE, with individuals with HF showing a higher prevalence of DE than those without HF (Multimedia Appendix 5). Moreover, there was a positive correlation between DE severity and HF symptoms (Tables 1 and 2). HF was initially diagnosed and treated by nonophthalmology clinicians. Furthermore, a system that allows ocular specialists to manage common ophthalmic comorbidities, including DE and allergic conjunctivitis, may facilitate the early prevention of chronic ocular damage.

DE and HF cause a wide range of symptoms, and their management is complicated by the wide variation in the presenting symptoms across individuals [13,14,17-19]. Allergic conjunctivitis, which is frequently observed in HF, has numerous common clinical features and subjective descriptions as DE [9]; therefore, careful evaluation is necessary. We used a dimension reduction algorithm to stratify DE and HF into clusters based on the ePROs collected using AllerSearch. We identified 14 unique clusters using this UMAP dimension reduction strategy, which successfully distinguished patterns in the presentations of DE, HF, and their comorbid state. Cluster 1, shown in Figures 1D and 1E, strongly exhibited symptoms of both DE and HF as well as worse QoL indicators compared with the other clusters. In contrast, cluster 9 exhibited minimal DE-related symptoms, with higher scores related to HF. The characteristics of each cluster demonstrated the proclivity of each patient to certain symptoms. Furthermore, a targeted approach based on early identification through stratification strategies using smartphone app–based data may allow improvement of long-term outcomes of HF management.

Although we observed a positive correlation between the individual symptoms of DE and HF, ocular grittiness and sore eyes (J-OSDI 2 and 3) showed a relatively stronger correlation with eye itchiness, tearing, and redness (NNSS 1, 2, and 3). Three environmental trigger–related items of J-OSDI regarding wind, humidity, and air conditioning (J-OSDI 10, 11, and 12) correlated with 2 NNSS items regarding eye itchiness and tearing (NNSS 1 and 2), which could exacerbate the risks of environmental factors. Five J-OSDI items regarding light sensitivity, blurred vision, poor vision, difficulty driving at night, and difficulty working with computers or automatic teller machines (J-OSDI 1, 4, 5, 7, and 8) appeared to distinguish individuals with HF and comorbid DE.

This study identified several factors and characteristics associated with comorbid DE among individuals with HF (Table 3), most of which have been previously reported as risk factors for DE [55,56]. Our results do not conclusively show a pathophysiological link between DE and inflammatory responses, including atopic dermatitis, food allergies, predisposition to ocular inflammation, and disruption of tear homeostasis, among patients with an increased lifetime likelihood of developing chronic allergic ocular surface diseases [57-60]. This might ultimately contribute to the development of DE in these populations. CL discontinuation is another characteristic of patients with comorbid DE and HF. Both diseases have been reported as common causes of CL discontinuation; however, their comorbidity is yet to be proposed as a marker [16]. Our findings suggest that careful evaluation of certain factors and individual characteristics may facilitate the prompt diagnosis and management of comorbid DE and HF, which could prevent long-term end-organ damage caused by chronic inflammatory states.

### Limitations

This crowdsourced clinical study has some limitations, as previously reported [13,14]. Our findings may be affected by selection bias for age, socioeconomic factors, health-seeking behavior, and characteristics of iPhone users in Japan. Moreover, we used self-administered questionnaires, which could have led to a recall bias and symptom overreporting. Nonetheless, the J-OSDI and JACQLQ have been validated through a comparison between paper-based and app-based questionnaires [11,34], which suggests that the results of other self-administered questionnaires are reliable. Finally, we determined the presence of DE and HF solely based on the questionnaires, which increased the possibility of false positives because physician-led clinical examination was not required. Owing to the limitations associated with the mHealth-based study, the external validity or generalizability of the findings of this study was also limited [12,14].

### Conclusions

This study demonstrated the epidemiology of comorbid symptomatic DE and HF as well as their exacerbating effects on the other and related factors that may indicate or contribute to their comorbidity. In addition, our findings demonstrated the utility of symptom-based stratification techniques for identifying subgroups of individual diseases as well as risk factors and characteristics unique to symptomatic DE, HF, and their comorbid state. Using the symptom-based stratification strategy could allow early identification of an individual’s associated subgroup and the underlying pathology. This may in turn facilitate efficient ocular care for individuals with DE and HF as well as inform the development of precision and preventative medicine for highly heterogeneous diseases with various comorbidities, which may obscure the prompt diagnosis of chronic disabling diseases.

## Appendix

Multimedia Appendix 1 Survey questions.

Multimedia Appendix 2 Subjective questionnaire for daily symptoms of hay fever.

Multimedia Appendix 3 Dry eye symptoms in the non–hay fever and hay fever groups.

Multimedia Appendix 4 Quality of life questionnaires for hay fever based on the Japanese Allergic Conjunctival Disease Quality of Life Questionnaire.

Multimedia Appendix 5 Characteristics of the study participants.

Multimedia Appendix 6 Symptoms and quality of life of dry eye and hay fever in each stratified cluster.

## Abbreviations

CL: contact lens
ClinRO: clinician-reported outcome
DE: dry eye
ePRO: electronic patient-reported outcome
HF: hay fever
JACQLQ: Japanese Allergic Conjunctival Disease Quality of Life Questionnaire
J-OSDI: Japanese version of the Ocular Surface Disease Index
mHealth: mobile health
NNSS: nonnasal symptom score
NSS: nasal symptom score
OR: odds ratio
PRO: patient-reported outcome
QoL: quality of life
TSS: total symptom score
UMAP: Uniform Manifold Approximation and Projection

## Data Availability

The codes that support the findings of this study are available from the corresponding author upon reasonable request.

## References

[1] Craig JP, Nichols KK, Akpek EK, Caffery B, Dua HS, Joo CK, Liu Z, Nelson JD, Nichols JJ, Tsubota K, Stapleton F (2017). TFOS DEWS II definition and classification report. Ocul Surf.

[2] Tsubota K, Yokoi N, Shimazaki J, Watanabe H, Dogru M, Yamada M, Kinoshita S, Kim HM, Tchah HW, Hyon JY, Yoon KC, Seo KY, Sun X, Chen W, Liang L, Li M, Liu Z, Asia Dry Eye Society (2017). New perspectives on dry eye definition and diagnosis: a consensus report by the Asia Dry Eye Society. Ocul Surf.

[3] Uchino M, Schaumberg DA (2013). Dry eye disease: impact on quality of life and vision. Curr Ophthalmol Rep.

[4] Yamada M, Mizuno Y, Shigeyasu C (2012). Impact of dry eye on work productivity. Clinicoecon Outcomes Res.

[5] Palmares J, Delgado L, Cidade M, Quadrado MJ, Filipe HP, Season Study Group (2010). Allergic conjunctivitis: a national cross-sectional study of clinical characteristics and quality of life. Eur J Ophthalmol.

[6] de la Hoz Caballer B, Rodríguez M, Fraj J, Cerecedo I, Antolín-Amérigo D, Colás C (2012). Allergic rhinitis and its impact on work productivity in primary care practice and a comparison with other common diseases: the Cross-sectional study to evAluate work Productivity in allergic Rhinitis compared with other common dIseases (CAPRI) study. Am J Rhinol Allergy.

[7] Leonardi A, Modugno RL, Salami E (2021). Allergy and dry eye disease. Ocul Immunol Inflamm.

[8] Ayaki M, Kawashima M, Uchino M, Tsubota K, Negishi K (2017). Possible association between subtypes of dry eye disease and seasonal variation. Clin Ophthalmol.

[9] Hom MM, Nguyen AL, Bielory L (2012). Allergic conjunctivitis and dry eye syndrome. Ann Allergy Asthma Immunol.

[10] Kumar N, Feuer W, Lanza NL, Galor A (2015). Seasonal variation in dry eye. Ophthalmology.

[11] Inomata T, Nakamura M, Iwagami M, Shiang T, Yoshimura Y, Fujimoto K, Okumura Y, Eguchi A, Iwata N, Miura M, Hori S, Hiratsuka Y, Uchino M, Tsubota K, Dana R, Murakami A (2019). Risk factors for severe dry eye disease: crowdsourced research using DryEyeRhythm. Ophthalmology.

[12] Inomata T, Iwagami M, Nakamura M, Shiang T, Yoshimura Y, Fujimoto K, Okumura Y, Eguchi A, Iwata N, Miura M, Hori S, Hiratsuka Y, Uchino M, Tsubota K, Dana R, Murakami A (2020). Characteristics and risk factors associated with diagnosed and undiagnosed symptomatic dry eye using a smartphone application. JAMA Ophthalmol.

[13] Inomata T, Nakamura M, Iwagami M, Sung J, Nakamura M, Ebihara N, Fujisawa K, Muto K, Nojiri S, Ide T, Okano M, Okumura Y, Fujio K, Fujimoto K, Nagao M, Hirosawa K, Akasaki Y, Murakami A (2021). Symptom-based stratification for hay fever: a crowdsourced study using the smartphone application AllerSearch. Allergy.

[14] Inomata T, Nakamura M, Sung J, Midorikawa-Inomata A, Iwagami M, Fujio K, Akasaki Y, Okumura Y, Fujimoto K, Eguchi A, Miura M, Nagino K, Shokirova H, Zhu J, Kuwahara M, Hirosawa K, Dana R, Murakami A (2021). Smartphone-based digital phenotyping for dry eye toward P4 medicine: a crowdsourced cross-sectional study. NPJ Digit Med.

[15] Dumbleton K, Caffery B, Dogru M, Hickson-Curran S, Kern J, Kojima T, Morgan PB, Purslow C, Robertson DM, Nelson JD, members of the TFOS International Workshop on Contact Lens Discomfort (2013). The TFOS international workshop on contact lens discomfort: report of the subcommittee on epidemiology. Invest Ophthalmol Vis Sci.

[16] Inomata T, Nakamura M, Iwagami M, Midorikawa-Inomata A, Sung J, Fujimoto K, Okumura Y, Eguchi A, Iwata N, Miura M, Fujio K, Nagino K, Hori S, Tsubota K, Dana R, Murakami A (2020). Stratification of individual symptoms of contact lens-associated dry eye using the iPhone app DryEyeRhythm: crowdsourced cross-sectional study. J Med Internet Res.

[17] Inomata T, Sung J, Nakamura M, Fujisawa K, Muto K, Ebihara N, Iwagami M, Nakamura M, Fujio K, Okumura Y, Okano M, Murakami A (2020). New medical big data for P4 medicine on allergic conjunctivitis. Allergol Int.

[18] Inomata T, Sung J, Nakamura M, Iwagami M, Okumura Y, Iwata N, Midorikawa-Inomata A, Fujimoto K, Eguchi A, Nagino K, Fujio K, Miura M, Shokirova H, Murakami A (2020). Using medical big data to develop personalized medicine for dry eye disease. Cornea.

[19] Inomata T, Sung J, Nakamura M, Iwagami M, Okumura Y, Fujio K, Akasaki Y, Fujimoto K, Yanagawa A, Midorikawa-Inomata A, Nagino K, Eguchi A, Shokirova H, Zhu J, Miura M, Kuwahara M, Hirosawa K, Huang T, Morooka Y, Murakami A (2021). Cross-hierarchical integrative research network for heterogenetic eye disease toward P4 medicine: a narrative review. Juntendo Med J.

[20] Weldring T, Smith SM (2013). Patient-Reported Outcomes (PROs) and Patient-Reported Outcome Measures (PROMs). Health Serv Insights.

[21] U.S. Department of Health and Human Services FDA Center for Drug Evaluation and Research, U.S. Department of Health and Human Services FDA Center for Biologics Evaluation and Research, U.S. Department of Health and Human Services FDA Center for Devices and Radiological Health (2006). Guidance for industry: patient-reported outcome measures: use in medical product development to support labeling claims: draft guidance. Health Qual Life Outcomes.

[22] Mayo NE, Figueiredo S, Ahmed S, Bartlett SJ (2017). Montreal accord on Patient-Reported Outcomes (PROs) use series - paper 2: terminology proposed to measure what matters in health. J Clin Epidemiol.

[23] Sawatzky R, Kwon JY, Barclay R, Chauhan C, Frank L, van den Hout WB, Nielsen LK, Nolte S, Sprangers MA, Response Shift – in Sync Working Group (2021). Implications of response shift for micro-, meso-, and macro-level healthcare decision-making using results of patient-reported outcome measures. Qual Life Res.

[24] Miljanović B, Dana R, Sullivan DA, Schaumberg DA (2007). Impact of dry eye syndrome on vision-related quality of life. Am J Ophthalmol.

[25] (2020). Modes of transmission of virus causing COVID-19: implications for IPC precaution recommendations. World Health Organization.

[26] Basch E, Iasonos A, McDonough T, Barz A, Culkin A, Kris MG, Scher HI, Schrag D (2006). Patient versus clinician symptom reporting using the national cancer institute common terminology criteria for adverse events: results of a questionnaire-based study. Lancet Oncol.

[27] Acevedo-Navarro L, Alvelo J, Torres B (1992). Activities of daily functioning and community adjustment of veterans in the VA residential care program. P R Health Sci J.

[28] Hua R, Yao K, Hu Y, Chen L (2014). Discrepancy between subjectively reported symptoms and objectively measured clinical findings in dry eye: a population based analysis. BMJ Open.

[29] LeBlanc TW, Abernethy AP (2017). Patient-reported outcomes in cancer care - hearing the patient voice at greater volume. Nat Rev Clin Oncol.

[30] Inomata T, Iwagami M, Nakamura M, Shiang T, Fujimoto K, Okumura Y, Iwata N, Fujio K, Hiratsuka Y, Hori S, Tsubota K, Dana R, Murakami A (2020). Association between dry eye and depressive symptoms: large-scale crowdsourced research using the DryEyeRhythm iPhone application. Ocul Surf.

[31] Eberle C, Löhnert M, Stichling S (2021). Effectiveness of disease-specific mHealth apps in patients with diabetes mellitus: scoping review. JMIR Mhealth Uhealth.

[32] Inomata T, Nakamura M, Iwagami M, Sung J, Nakamura M, Ebihara N, Fujisawa K, Muto K, Nojiri S, Ide T, Okano M, Okumura Y, Fujio K, Fujimoto K, Nagao M, Hirosawa K, Akasaki Y, Murakami A (2022). Individual characteristics and associated factors of hay fever: a large-scale mHealth study using AllerSearch. Allergol Int.

[33] Kirtsreesakul V, Somjareonwattana P, Ruttanaphol S (2009). The correlation between nasal symptom and mucociliary clearance in allergic rhinitis. Laryngoscope.

[34] Akasaki Y, Inomata T, Sung J, Okumura Y, Fujio K, Miura M, Hirosawa K, Iwagami M, Nakamura M, Ebihara N, Nakamura M, Ide T, Nagino K, Murakami A (2022). Reliability and validity of electronic patient-reported outcomes using the smartphone app AllerSearch for hay fever: prospective observational study. JMIR Form Res.

[35] Akasaki Y, Inomata T, Sung J, Nakamura M, Kitazawa K, Shih KC, Adachi T, Okumura Y, Fujio K, Nagino K, Midorikawa-Inomata A, Kuwahara M, Hirosawa K, Huang T, Morooka Y, Shokirova H, Eguchi A, Murakami A (2022). Prevalence of comorbidity between dry eye and allergic conjunctivitis: a systematic review and meta-analysis. J Clin Med.

[36] Midorikawa-Inomata A, Inomata T, Nojiri S, Nakamura M, Iwagami M, Fujimoto K, Okumura Y, Iwata N, Eguchi A, Hasegawa H, Kinouchi H, Murakami A, Kobayashi H (2019). Reliability and validity of the Japanese version of the Ocular Surface Disease Index for dry eye disease. BMJ Open.

[37] Fukagawa K, Fujishima H, Fukushima A, Sumi T, Okamoto S, Shoji J, Satake Y, Ohno S, Namba K, Kitaichi N, Ebihara N, Takahashi H, Kumagai N, Uchino Y, Uchino M, Murayama K, Sakata M, Uchio E, Takamura E, Ohashi Y, Ohkubo K, Satoh T (2012). [A quality of life questionnaire for Japanese allergic conjunctival disease]. Nippon Ganka Gakkai Zasshi.

[38] Fujio K, Inomata T, Fujisawa K, Sung J, Nakamura M, Iwagami M, Muto K, Ebihara N, Nakamura M, Okano M, Akasaki Y, Okumura Y, Ide T, Nojiri S, Nagao M, Fujimoto K, Hirosawa K, Murakami A (2022). Patient and public involvement in mobile health-based research for hay fever: a qualitative study of patient and public involvement implementation process. Res Involv Engagem.

[39] Schiffman RM, Christianson MD, Jacobsen G, Hirsch JD, Reis BL (2000). Reliability and validity of the Ocular Surface Disease Index. Arch Ophthalmol.

[40] Miller KL, Walt JG, Mink DR, Satram-Hoang S, Wilson SE, Perry HD, Asbell PA, Pflugfelder SC (2010). Minimal clinically important difference for the ocular surface disease index. Arch Ophthalmol.

[41] von Luxburg U (2007). A tutorial on spectral clustering. Stat Comput.

[42] McInnes L, Healy J, Saul N, Großberger L (2018). UMAP: uniform manifold approximation and projection. J Open Source Softw.

[43] Mann HB, Whitney DR (1947). On a test of whether one of two random variables is stochastically larger than the other. Ann Math Stat.

[44] Kruskal WH, Wallis WA (1952). Use of ranks in one-criterion variance analysis. J Am Stat Assoc.

[45] McHugh ML (2013). The chi-square test of independence. Biochem Med (Zagreb).

[46] Brown CE, Brown CE (1998). Multiple logistic regression. Applied Multivariate Statistics in Geohydrology and Related Sciences.

[47] Schober P, Boer C, Schwarte LA (2018). Correlation coefficients: appropriate use and interpretation. Anesth Analg.

[48] Jobson JD, Jobson JD (1991). Multiple linear regression. Applied Multivariate Data Analysis: Regression and Experimental Design.

[49] Peuravuori H, Kari O, Peltonen S, Aho VV, Saari JM, Collan Y, Määttä M, Saari KM (2004). Group IIA phospholipase A2 content of tears in patients with atopic blepharoconjunctivitis. Graefes Arch Clin Exp Ophthalmol.

[50] Suzuki S, Goto E, Dogru M, Asano-Kato N, Matsumoto Y, Hara Y, Fujishima H, Tsubota K (2006). Tear film lipid layer alterations in allergic conjunctivitis. Cornea.

[51] Malu KN (2014). Allergic conjunctivitis in Jos-Nigeria. Niger Med J.

[52] Akil H, Celik F, Ulas F, Kara IS (2015). Dry eye syndrome and allergic conjunctivitis in the pediatric population. Middle East Afr J Ophthalmol.

[53] Chen L, Pi L, Fang J, Chen X, Ke N, Liu Q (2016). High incidence of dry eye in young children with allergic conjunctivitis in Southwest China. Acta Ophthalmol.

[54] Meltzer EO, Blaiss MS, Derebery MJ, Mahr TA, Gordon BR, Sheth KK, Simmons AL, Wingertzahn MA, Boyle JM (2009). Burden of allergic rhinitis: results from the pediatric allergies in America survey. J Allergy Clin Immunol.

[55] Stapleton F, Alves M, Bunya VY, Jalbert I, Lekhanont K, Malet F, Na KS, Schaumberg D, Uchino M, Vehof J, Viso E, Vitale S, Jones L (2017). TFOS DEWS II epidemiology report. Ocul Surf.

[56] Yamanishi R, Sawada N, Hanyuda A, Uchino M, Kawashima M, Yuki K, Tsubota K, Kato T, Saito I, Arima K, Mizukami S, Tanno K, Sakata K, Yamagishi K, Iso H, Yasuda N, Shimazu T, Yamaji T, Goto A, Inoue M, Iwasaki M, Tsugane S, JPHC-NEXT Group (2021). Relation between body mass index and dry eye disease: the Japan public health center-based prospective study for the next generation. Eye Contact Lens.

[57] Onguchi T, Dogru M, Okada N, Kato NA, Tanaka M, Takano Y, Fukagawa K, Shimazaki J, Tsubota K, Fujishima H (2006). The impact of the onset time of atopic keratoconjunctivitis on the tear function and ocular surface findings. Am J Ophthalmol.

[58] Dogru M, Asano-Kato N, Tanaka M, Igarashi A, Shimmura S, Shimazaki J, Okada N, Takano Y, Fukagawa K, Tsubota K, Fujishima H (2005). Ocular surface and MUC5AC alterations in atopic patients with corneal shield ulcers. Curr Eye Res.

[59] Toda I, Shimazaki J, Tsubota K (1995). Dry eye with only decreased tear break-up time is sometimes associated with allergic conjunctivitis. Ophthalmology.

[60] Hu Y, Matsumoto Y, Dogru M, Okada N, Igarashi A, Fukagawa K, Tsubota K, Fujishima H (2007). The differences of tear function and ocular surface findings in patients with atopic keratoconjunctivitis and vernal keratoconjunctivitis. Allergy.

