# Supplementary material for: Using the AllerSearch Smartphone App to Assess the Association Between Dry Eye and Hay Fever: mHealth-Based Cross-Sectional Study
Source: J Med Internet Res. 2023 Sep 12;25:e38481. doi: 10.2196/38481 (PMC10523221; doi:10.2196/38481)
Supplement: Multimedia Appendix 1 [file jmir_v25i1e38481_app1.docx]

**Multimedia Appendix 1.** Survey questions.

| **Questions** | **Variables** | **Details of variables** |
| --- | --- | --- |
| **User characteristics** | | |
|  | Age | Integer input, years |
|  | Sex | Choose one {'Man,' 'Woman'} |
|  | Height | Integer input, cm^a^ |
|  | Weight | Integer input, kg^b^ |
| Please provide information regarding your siblings.  How many siblings do you have?  What is your position among your siblings (e.g., first-born, second-born)? | Sibling | Integer input, sibling count, sibling position |
| **Medical history** | | |
| Have you ever been diagnosed with hypertension? | Medicated hypertension | Choose one {'No,' 'I am being treated for hypertension,' 'I have untreated hypertension,' ' I do not know'} |
| Have you ever been diagnosed with diabetes? | Diabetes (HbA1c^c^ level) | Choose one {'Yes,' 'No,' ' I do not know'}. If 'Yes,’ scale bar input of HbA1c level (5–15%) |
| Have you experienced any of the following illnesses? | Systemic diseases | Multiple choice among {'Heart disease,' 'Respiratory disease,' 'Brain disease,' 'Liver disease,' 'Kidney disease,' 'Blood disease,' 'Malignant tumor,' 'Collagen disease,' 'N/A^d^'} |
| If you have eczema (atopic dermatitis), when did it start? | Atopic dermatitis | Choose one {'Infancy (before 1 year of age),' 'Early childhood (age 1–6 years),' 'Middle childhood (age 7–12 years),' Early adolescence (after the age of 13 years)'} |
| Do you experience swollen lips after consuming products containing tomato? | Tomato allergy | Choose one {'Yes,' 'No'} |
| Do you have any mental illness? | Mental illness | Choose one {'No,' 'Yes,' 'Previously had'} |
| Do you have any of the following mental illnesses? | Mental illness | If 'Yes’ in the previous question, multiple choice among {'Depression,' 'Schizophrenia,' 'Other mental illness'} |
| Have you ever been diagnosed with dry eye disease? | Dry eye disease | Choose one {'No,' 'Yes,' 'I do not know'} |
| **Residential environment** | | |
| What type of flooring do you have in your living room? | Living | Choose one {'Hardwood,' 'Carpet,' 'Tatami (Japanese straw-based floor),' 'Vinyl,' 'Other'} |
| What type of flooring do you have in your bedroom? | Bedroom | Choose one {'Hardwood,' 'Carpet,' 'Tatami (Japanese straw-based floor),' 'Vinyl,' 'Other'} |
| Do you currently own any pets? | Pets | Multiple choice among {'No,' Dog,' 'Cat,' 'Rabbit,' 'Rodents (such as hamsters, guinea pigs),' 'Birds,' 'Other'} |
| **Lifestyle** | | |
| How many cups of coffee do you drink per day on average? | Coffee intake | Integer input, cups |
| Have you ever used contact lenses? | Contact lens use | Choose one {'I have been using contact lenses,' 'I have, but they were discontinued during hay fever season,' 'I have used contact lenses in the past,' 'I have never used contact lenses'} |
| What type of contact lenses do (or have) you use(d)? | Types of contact lenses | Choose one {'Soft/Daily disposable,' 'Soft/Bi-weekly disposable,' 'Soft/Monthly disposable,' 'Soft/Yearly disposable,' 'Hard,' 'Colored/Daily disposable,' 'Colored/Bi-weekly disposable,' 'Colored/Monthly disposable,' 'Colored/Yearly disposable'} |
| Please enter your exercise frequency and duration in the past week. | Exercise | Integer input, days and hours |
| Please select the major exercise type that you undertake. | Exercise category | Choose one {'Walking,' 'Light exercise,' 'Bowling,' 'Swimming,' 'Equipment-based training,' 'Jogging,' 'Marathon,' 'Hiking,' 'Cycling,' 'Fishing,' 'Golfing (including indoor ranges),' 'Baseball (including catch ball),' 'Other'} |
| Are you using eye drops (artificial tears)? | Eye drops | Choose one {'Currently using (non-contact lens user),' 'Currently using without removing contact lens (contact lens user),' 'Currently using after removing contact lenses (contact lens user),' 'Not currently using'} |
| Do you use eye wash solutions? If yes, at what time of the day do you use them? | Eye wash | Multiple choice among {'Morning,' 'Afternoon,' 'Evening,' 'Before sleeping,' 'When symptoms worsen,' 'Do not use eye wash'} |
| Please enter the average frequency of bowel movements per week. | Bowel movements | Integer input, times |
| Please enter your average sleep duration per day. | Sleep duration | Integer input, hours |
| Do you currently smoke or have you smoked in the past? | Smoking | Choose one {'No,' 'Yes,' 'Have before'} If 'Yes’ or ‘Have before,’ scale bar input of Number of cigarettes per day, Number of years smoked |
| How many times do you consume yogurt (or yogurt-containing products) per week? | Yogurt intake | Choose one {'Rarely,' 'Once a week,' 'Twice or thrice a week,' 'Four or five times a week,' 'Everyday'} |
| **Hay fever** | | |
| Do you have hay fever? | Hay fever | Choose one {'Yes,' 'No,' 'Unknown'} |
| When did your hay fever start? | Hay fever onset | Integer input, years old |
| In which months of the year does your hay fever occur? | Months when hay fever develops | Multiple choice among {'January,' 'February,' 'March,' 'April,' 'May,' 'June,' 'July,' 'August,' 'September,' 'October,' 'November,' 'December'} |
| In which months of the year does your hay fever worsen? | Months when hay fever worsens | Multiple choice among {'January,' 'February,' 'March,' 'April,' 'May,' 'June,' 'July,' 'August,' 'September,' 'October,' 'November,' 'December'} |
| Have you ever received subcutaneous or sublingual allergen immunotherapy (desensitization/hyposensitization)? | Sublingual and subcutaneous desensitization therapy | Choose one {'Yes,' 'No'} |
| Please tell us which of the following hay fever prevention methods you have been using. | Preventive behavior | Multiple choice among {'Mask,' 'Eye drops,' 'Nasal spray/drops,' 'Medication,' 'Air purifier,' 'Glasses and goggles,' 'Other,' 'Not using any'} |

^a^cm: centimeter.

^b^HbA1c: glycated hemoglobin A1c.

^c^kg: kilogram.

^d^N/A: not applicable.
