# Supplementary material for: Using the AllerSearch Smartphone App to Assess the Association Between Dry Eye and Hay Fever: mHealth-Based Cross-Sectional Study
Source: J Med Internet Res. 2023 Sep 12;25:e38481. doi: 10.2196/38481 (PMC10523221; doi:10.2196/38481)
Supplement: Multimedia Appendix 2 [file jmir_v25i1e38481_app2.docx]

**Multimedia Appendix 2.** Subjective questionnaire for daily symptoms of hay fever.

| **Questions** | **Variables** | **Details of variables** |
| --- | --- | --- |
| **Nasal symptom score** | | Choose one {'No symptoms, ' 'Mild symptoms (symptoms clearly present but easily tolerated),' 'Moderate symptoms (symptoms bothersome but tolerable),' 'Severe symptoms (symptoms difficult to tolerate—interfere with activities)'} |
| Please rate how your rhinorrhea has been over the past 24 hours? | NSS^a^ item 1 |  |
| Please rate how your nasal congestion has been over the past 24 hours? | NSS item 2 |  |
| Please rate how your nasal itching has been over the past 24 hours? | NSS item 3 |  |
| Please rate how your sneezing has been over the past 24 hours? | NSS item 4 |  |
| How severely does hay fever affect your daily activities? | NSS item 5 |  |
| **Nonnasal symptom score** | |  |
| How much eye itching did you experience over the past 24 hours? | NNSS^b^ item 1 |  |
| How much eye-watering did you experience over the past 24 hours? | NNSS item 2 |  |
| How much eye redness did you experience over the past 24 hours? | NNSS item 3 |  |
| How much itching of the ear and nose did you experience over the past 24 hours? | NNSS item 4 |  |

^a^NSS: nasal symptom score.

^b^NNSS: nonnasal symptom score.
