# Supplementary material for: Using the AllerSearch Smartphone App to Assess the Association Between Dry Eye and Hay Fever: mHealth-Based Cross-Sectional Study
Source: J Med Internet Res. 2023 Sep 12;25:e38481. doi: 10.2196/38481 (PMC10523221; doi:10.2196/38481)
Supplement: Multimedia Appendix 3 [file jmir_v25i1e38481_app3.docx]

**Multimedia Appendix 3.** Dry eye symptoms in the non-hay fever and hay fever groups (n=11,284).^a^

|  | Non-HF^b^ (n=720),  DE^c^ symptom status | | | HF (n=9041),  DE symptom status | | | Unknown (n=1523),  DE symptom status | | |  |
| --- | --- | --- | --- | --- | --- | --- | --- | --- | --- | --- |
|  | Nonsymptomatic DE (n=  452) | Symptomatic DE  (n=268) | *P* value | Nonsymptomatic DE  (n=4612) | Symptomatic DE  (n=4429) | *P* value | Nonsymptomatic DE  (n=780) | Symptomatic DE  (n=743) | *P* value |  |
| **J-OSDI^d^ questionnaire, items, 0-4, median [IQR^e^]** | | | | | | | | | | |
| 1. Eyes that are sensitive to light? | 0 (0-1) | 1 (1-2) | <.001 | 0 (0-1) | 1 (1-2) | <.001 | 0 (0-1) | 1 (1-2) | <.001 |  |
| 2. Eyes that feel gritty? | 0 (0-1) | 1 (1-1) | <.001 | 0 (0-1) | 1 (1-2) | <.001 | 0 (0-1) | 1 (1-2) | <.001 |  |
| 3. Painful or sore eyes? | 0 (0-0) | 1 (0-1) | <.001 | 0 (0-0) | 1 (0-1) | <.001 | 0 (0-0) | 1 (0-1) | <.001 |  |
| 4. Blurred vision? | 0 (0-0) | 1 (0-1) | <.001 | 0 (0-1) | 1 (1-1) | <.001 | 0 (0-1) | 1 (1-1) | <.001 |  |
| 5. Poor vision? | 0 (0-0) | 1 (1-1) | <.001 | 0 (0-0) | 1 (1-1) | <.001 | 0 (0-0) | 1 (0-1) | <.001 |  |
| 6. Reading? | 0 (0-0) | 1 (0-1) | <.001 | 0 (0-0) | 1 (0-1) | <.001 | 0 (0-0) | 1 (0-1) | <.001 |  |
| 7. Driving at night? | 0 (0-0) | 1 (0-3) | <.001 | 0 (0-0) | 1 (0-5) | <.001 | 0 (0-0) | 1 (0-4) | <.001 |  |
| 8. Working with a computer or bank machine (ATM^f^)? | 0 (0-0) | 1 (0-1) | <.001 | 0 (0-0) | 0 (0-1) | <.001 | 0 (0-0) | 0 (0-1) | <.001 |  |
| 9. Watching TV^g^? | 0 (0-0) | 1 (0-1) | <.001 | 0 (0-0) | 1 (0-1) | <.001 | 0 (0-0) | 0 (0-1) | <.001 |  |
| 10. Windy conditions? | 0 (0-0) | 1 (0-1) | <.001 | 0 (0-0) | 1 (0-2) | <.001 | 0 (0-0) | 1 (0-2) | <.001 |  |
| 11. Places or areas with low humidity (very dry)? | 0 (0-0) | 1 (1-2) | <.001 | 0 (0-1) | 1 (1-3) | <.001 | 0 (0-1) | 1 (1-3) | <.001 |  |
| 12. Areas that are air-conditioned? | 0 (0-0) | 1 (1-2) | <.001 | 0 (0-1) | 1 (1-3) | <.001 | 0 (0-1) | 1 (1-3) | <.001 |  |
| J-OSDI total score, 0-100, median [IQR] | 4.2 (0-8.3) | 22.9 (16.7-31.5) | <.001 | 6.3 (2.1-9.1) | 25 (18.2-33.3) | <.001 | 6.3 (2.1-10.4) | 22.9 (17.5-22.9) | <.001 |  |
| Ocular symptoms, items 1-5, 0-100, median [IQR] | 5 (0-10) | 10 (5-20) | <.001 | 5 (0-12.5) | 25 (20-35) | <.001 | 5 (0-15) | 25 (20-35) | <.001 |  |
| Vision-related function, items 6-9, 0-100, median [IQR] | 0 (0-0) | 18.8 (6.3-25) | <.001 | 0 (0-0) | 12.5 (6.3-25) | <.001 | 0 (0-0) | 12.5 (6.3-25) | <.001 |  |
| Environmental triggers, 0-100, items 10-12, median [IQR] | 0 (0-8.3) | 25 (16.7-50) | <.001 | 0 (0-16.7) | 25 (16.7-50) | <.001 | 0 (0-16.7) | 25 (16.7-50) | <.001 |  |

^a^Mann–Whitney U tests were performed for continuous variables. Statistical significance was set at *P*<.05.

^b^HF: hay fever.

^c^DE: dry eye.

^d^J-OSDI: Japanese version of Ocular Surface Disease Index.

^e^IQR: interquartile range.

^f^ATM: automatic teller machine.

^g^TV: television.
