# Supplementary material for: Using the AllerSearch Smartphone App to Assess the Association Between Dry Eye and Hay Fever: mHealth-Based Cross-Sectional Study
Source: J Med Internet Res. 2023 Sep 12;25:e38481. doi: 10.2196/38481 (PMC10523221; doi:10.2196/38481)
Supplement: Multimedia Appendix 4 [file jmir_v25i1e38481_app4.docx]

**Multimedia Appendix 4.** Quality of life questionnaires for hay fever based on the JACQLQ^a^.

| **JACQLQ items** | **Detail of variables** |
| --- | --- |
| **Domain I, 0–36** | |
| In the previous 1–2 weeks, have you had following eye and/or nose symptoms? | Choose one {'zero to four’ with lower scores reflecting lower frequency of eye and nose symptoms} |
| 1. Itchy eyes |  |
| 2. Foreign body sensation |  |
| 3. Red eyes |  |
| 4. Watery eyes |  |
| 5. Eye discharge |  |
| 6. Runny nose |  |
| 7. Sneezing |  |
| 8.Stuffy nose |  |
| 9. Itchy nose |  |
| **Domain II, 0–68** | |
| In the previous 1–2 weeks, has your life been interrupted by eye and/or nose symptoms? | Choose one {'zero to four’ with lower scores reflecting better health-related QoL^b^} |
| 1. Reduced productivity at work/school/home |  |
| 2. Poor mental concentration |  |
| 3. Reduced thinking power |  |
| 4. Impaired reading |  |
| 5. Poor memory |  |
| 6. Limitation of outdoor life |  |
| 7. Limitation of going out |  |
| 8. Reluctance to visit friends |  |
| 9. Reduced contact with friends |  |
| 10. Uneasy with people around you |  |
| 11. Impaired sleeping |  |
| 12. Tiredness |  |
| 13. Fatigue |  |
| 14. Frustrated |  |
| 15. Irritable |  |
| 16. Depressed |  |
| 17. Unhappy |  |

^a^JACQLQ: Japanese Allergic Conjunctival Disease Standard QoL Questionnaire.

^b^QoL: quality of life.
