# Supplementary material for: Using the AllerSearch Smartphone App to Assess the Association Between Dry Eye and Hay Fever: mHealth-Based Cross-Sectional Study
Source: J Med Internet Res. 2023 Sep 12;25:e38481. doi: 10.2196/38481 (PMC10523221; doi:10.2196/38481)
Supplement: Multimedia Appendix 5 [file jmir_v25i1e38481_app5.docx]

**Multimedia Appendix 5.** Characteristics of the study participants (n=11,284).^a^

|  | | Non-HF^b^ (n=720),  DE^c^ symptom status | | | HF (n=9041),  DE symptom status | | | Unknown (n=1523),  DE symptom status | | | Total |
| --- | --- | --- | --- | --- | --- | --- | --- | --- | --- | --- | --- |
|  |  | Non symptomatic DE  (n=452 | Symptomatic DE  (n=268) | *P* value | Non symptomatic DE  (n=4612) | Symptomatic DE  (n=4429) | *P* value | Non symptomatic DE  (n=780) | Symptomatic DE  (n=743) | *P* value |  |
| **Demographic characteristics** | | | | | | | | | | | |
| Age (years), median (IQR^d^) |  | 38.5 (28-50) | 41 (27-52) | .413 | 34 (24-46) | 33 (23-46) | .083 | 32 (23-44) | 30 (22-44) | .104 | 34 (23-46) |
| Women, n (%) |  | 171 (37.8) | 139 (51.9) | <.001 | 2190 (47.5) | 2895 (65.4) | <.001 | 456 (58.5) | 507 (68.2) | <.001 | 6358 (56.4) |
| Body mass index, median (IQR) |  | 22.6 (20.0-25.4) | 22.1 (20.1-25.0) | .433 | 22.0 (19.9-24.4) | 21.5 (19.6-24.1) | <.001 | 21.7 (19.6-24.1) | 21.6 (19.7-23.9) | .459 | 21.8 (19.8-24.3) |
| **Medical history** | | | | | | | | | | | |
| Medicated hypertension, n (%) | No | 373 (82.5) | 205 (76.5) | .183 | 3815 (82.7) | 3515 (79.4) | <.001 | 603 (77.3) | 558 (75.1) | .625 | 9069 (80.4) |
|  | Medicated | 39 (8.6) | 26 (9.7) | — | 251 (5.4) | 242 (5.5) | — | 38 (4.9) | 35 (4.7) | — | 631 (5.6) |
|  | Unmedicated | 18 (4.0) | 17 (6.3) | — | 166 (3.6) | 145 (3.3) | — | 24 (3.1) | 22 (3.0) | — | 392 (3.5) |
|  | Unknown | 22 (4.9) | 20 (7.5) | — | 380 (8.2) | 527 (11.9) | — | 115 (14.7) | 128 (17.2) | — | 1192 (10.6) |
| Diabetes, n (%) | No | 409 (90.5) | 224 (83.6) | .017 | 4187 (90.8) | 3898 (88.0) | <.001 | 677 (86.8) | 624 (84.0) | .265 | 10019 (88.8) |
|  | Yes | 17 (3.8) | 21 (7.8) | — | 110 (2.4) | 91 (2.1) | — | 19 (2.4) | 10 (2.6) | — | 277 (2.5) |
|  | Unknown | 26 (5.8) | 23 (8.6) | — | 315 (6.8) | 440 (10.0) | — | 84 (10.8) | 100 (13.5) | — | 988 (8.8) |
| Systemic diseases, n (%) | Hematologic disease | 8 (1.8) | 8 (3.0) | .285 | 52 (1.1) | 83 (1.9) | .003 | 4 (0.5) | 10 (1.4) | .089 | 165 (1.5) |
|  | Brain disease | 7 (1.6) | 5 (1.9) | .748 | 44 (1.0) | 54 (1.2) | .223 | 8 (1.0) | 10 (1.4) | .563 | 128 (1.1) |
|  | Collagen disease | 3 (0.7) | 1 (0.4) | .612 | 14 (0.3) | 33 (0.8) | .004 | 5 (0.6) | 6 (0.8) | .701 | 62 (0.6) |
|  | Heart disease | 15 (3.3) | 9 (3.4) | .977 | 80 (1.7) | 101 (2.3) | .064 | 13 (1.7) | 25 (3.4) | .034 | 243 (2.2) |
|  | Kidney disease | 10 (2.2) | 3 (1.1) | .287 | 78 (1.7) | 84 (1.9) | .462 | 8 (1.0) | 14 (1.9) | .160 | 197 (1.8) |
|  | Liver disease | 3 (0.7) | 5 (1.9) | .137 | 62 (1.3) | 94 (2.1) | .005 | 7 (0.9) | 9 (1.2) | .548 | 180 (1.6) |
|  | Malignant tumor | 4 (0.9) | 9 (3.4) | .016 | 69 (1.5) | 60 (1.4) | .571 | 15 (1.9) | 8 (1.1) | .176 | 165 (1.5) |
|  | Respiratory disease | 38 (8.4) | 26 (9.7) | .555 | 351 (7.6) | 500 (11.3) | <.001 | 51 (6.5) | 61 (8.2) | .212 | 1027 (9.1) |
|  | N/A^e^ | 372 (82.3) | 214 (79.9) | .414 | 3922 (85.0) | 3567 (80.5) | <.001 | 678 (86.9) | 630 (84.8) | .232 | 9383 (83.2) |
| Atopic disease, n (%) |  | 56 (12.4) | 33 (12.3) | .976 | 732 (15.9) | 884 (20.0) | <.001 | 106 (13.6) | 117 (15.8) | .234 | 1928 (17.1) |
| Tomato allergy, n (%) |  | 8 (1.8) | 5 (1.9) | .926 | 87 (1.9) | 159 (3.6) | <.001 | 8 (1.0) | 20 (2.7) | .016 | 287 (2.5) |
| Mental illness, n (%) | No | 419 (92.7) | 235 (87.7) | .035 | 4243 (92.0) | 3759 (84.9) | <.001 | 697 (89.4) | 635 (85.5) | .072 | 9988 (88.5) |
|  | Yes | 15 (3.3) | 20 (7.5) | — | 151 (3.3) | 344 (7.8) | — | 40 (5.1) | 52 (7.0) | — | 622 (5.5) |
|  | Previously had | 18 (4.0) | 13 (4.9) | — | 218 (4.7) | 326 (7.4) | — | 43 (5.5) | 56 (7.5) | — | 674 (6.0) |
| History of dry eye diagnosis, n (%) | No | 322 (71.2) | 136 (50.8) | <.001 | 2996 (65.0) | 1673 (37.8) | <.001 | 465 (59.6) | 262 (35.3) | <.001 | 5854 (51.9) |
|  | Yes | 51 (11.3) | 82 (30.6) | — | 601 (13.0) | 1489 (33.6) | — | 86 (11.0) | 198 (26.7) | — | 2507 (22.2) |
|  | Unknown | 79 (17.5) | 50 (18.7) | — | 1015 (22.0) | 1267 (28.6) | — | 229 (29.4) | 283 (38.1) | — | 2923 (25.9) |
| **Residential environment** | | | | | | | | | | | |
| Living room, n (%) | Hardwood | 340 (75.2) | 181 (67.5) | .276 | 3386 (73.4) | 3160 (71.4) | .014 | 529 (67.8) | 485 (65.3) | .650 | 8081 (71.6) |
|  | Carpet | 82 (18.1) | 62 (23.1) | — | 866 (18.8) | 881 (19.9) | — | 173 (22.2) | 189 (25.4) | — | 2253 (20.0) |
|  | Tatami (Japanese straw–based floor) | 16 (3.5) | 14 (5.2) | — | 229 (5.0) | 245 (5.5) | — | 38 (4.9) | 36 (4.9) | — | 578 (5.1) |
|  | Vinyl | 5 (1.1) | 4 (1.5) | — | 70 (1.5) | 99 (2.2) | — | 25 (3.2) | 20 (2.7) | — | 223 (2.0) |
|  | Other | 9 (2.0) | 7 (2.6) | — | 61 (1.3) | 44 (1.0) | — | 15 (1.9) | 13 (1.8) | — | 149 (1.3) |
| Bedroom, n (%) | Hardwood | 295 (65.3) | 151 (56.3) | .093 | 2922 (63.4) | 2748 (62.1) | .288 | 471 (60.4) | 454 (61.1) | .011 | 7041 (62.4) |
|  | Carpet | 70 (15.5) | 50 (18.7) | — | 783 (17.0) | 771 (17.4) | — | 125 (16.0) | 147 (19.8) | — | 1946 (17.3) |
|  | Tatami (Japanese straw–based floor) | 73 (6.2) | 53 (19.8) | — | 803 (17.4) | 781 (17.6) | — | 140 (18.0) | 115 (15.5) | — | 1965 (17.4) |
|  | Vinyl | 3 (0.7) | 6 (2.2) | — | 52 (1.1) | 69 (1.6) | — | 20 (2.6) | 20 (1.7) | — | 170 (1.5) |
|  | Other | 11 (2.4) | 8 (3.0) | — | 52 (1.1) | 60 (1.4) | — | 24 (3.1) | 7 (0.9) | — | 162 (1.4) |
| Pet ownership, n (%) |  | 123 (27.2) | 99 (36.9) | .006 | 1368 (29.7) | 1606 (36.3) | <.001 | 273 (35.0) | 271 (36.5) | .549 | 3740 (33.1) |
| **Lifestyle habits** | | | | | | | | | | | |
| Coffee intake (cups per day), median (IQR) | | 1.5 (1-2.5) | 2 (1-3) | .210 | 1 (0-2) | 1 (0-2) | .027 | 1 (0-2) | 1 (0-2) | .090 | 1 (0-2) |
| Contact lens use, n (%) | No | 233 (51.6) | 130 (48.5) | .195 | 2296 (49.8) | 1829 (41.3) | <.001 | 387 (49.6) | 296 (39.8) | <.001 | 5171 (45.8) |
|  | Discontinued during the hay fever season | 94 (20.8) | 45 (16.8) | — | 814 (17.7) | 786 (17.8) | — | 134 (17.2) | 104 (14.0) | — | 1977 (17.5) |
|  | Past use | 4 (0.9) | 2 (0.8) | — | 112 (2.4) | 167 (3.8) | — | 3 (0.4) | 7 (0.9) | — | 295 (2.6) |
|  | Current use | 121 (26.8) | 91 (34.0) | — | 1390 (30.1) | 1647 (37.2) | — | 256 (32.8) | 336 (45.2) | — | 3841 (34.0) |
| Exercise (hours per week), median (IQR) | | 1 (0-2) | 1 (1-3) | .147 | 1 (0-2) | 1 (0-2) | .461 | 1 (0-2) | 1 (0-3) | .042 | 1 (0-2) |
| Exercise (days per week), median (IQR) | | 1 (0.5-3) | 1 (1-4) | .782 | 1 (0-4) | 1 (0-3) | .072 | 1 (0-4) | 2 (0-4) | .155 | 1 (0-4) |
| Bowel movements (times per week), median (IQR) | | 5 (2-7) | 5 (3-7) | .680 | 6 (3-7) | 6 (3-7) | .018 | 5 (3-7) | 5 (3-7) | .612 | 6 (3-7) |
| Sleep duration (hours per day), median (IQR) | | 6 (6-7) | 6 (6-7) | .107 | 6 (6-7) | 6 (6-7) | <.001 | 6 (6-7) | 6 (6-7) | .072 | 6 (6-7) |
|  | <6 | 85 (18.8) | 59 (22.0) | .097 | 843 (18.3) | 988 (22.3) | <.001 | 157 (20.1) | 569 (76.6) | .638 | 2295 (20.3) |
|  | 6–9 | 363 (80.3) | 202 (75.4) | — | 3717 (80.6) | 3384 (76.4) | — | 613 (78.6) | 163 (21.9) | — | 8848 (78.4) |
|  | >9 | 4 (0.9) | 7 (2.6) | — | 52 (1.1) | 57 (1.3) | — | 10 (1.3) | 11 (1.5) | — | 141 (1.3) |
| Smoking habit, n (%) | No | 308 (68.1) | 165 (61.6) | .129 | 3289 (71.3) | 3071 (69.3) | .001 | 555 (71.2) | 468 (63.0) | .003 | 7856 (69.6) |
|  | Yes | 74 (16.4) | 59 (22.0) | — | 606 (13.1) | 707 (16.0) | — | 121 (15.5) | 143 (19.3) | — | 1710 (15.2) |
|  | Previously had | 70 (15.5) | 44 (16.4) | — | 717 (15.6) | 651 (14.7) | — | 104 (13.3) | 132 (17.8) | — | 1718 (!5.2) |
| Yogurt intake, n (%) | Rarely | 195 (43.1) | 99 (36.9) | .062 | 1969 (42.7) | 1846 (41.7) | .569 | 382 (49.0) | 343 (46.2) | .627 | 4834 (42.8) |
|  | Once a week | 78 (17.3) | 65 (24.3) | — | 836 (18.1) | 807 (18.2) | — | 149 (19.1) | 147 (19.8) | — | 2082 (18.5) |
|  | Twice or thrice a week | 68 (15.0) | 49 (18.3) | — | 724 (15.7) | 724 (16.4) | — | 92 (11.8) | 103 (13.9) | — | 1760 (15.6) |
|  | 4 or 5 times a week | 41 (9.1) | 16 (6.0) | — | 359 (7.8) | 377 (8.5) | — | 59 (7.6) | 50 (6.7) | — | 902 (8.0) |
|  | Everyday | 70 (15.5) | 39 (14.6) | — | 724 (15.7) | 675 (15.2) | — | 98 (12.6) | 100 (13.5) | — | 1706 (15.1) |

^a^Mann-Whitney *U* tests were performed for continuous variables. The chi-square test was performed for categorical variables. Statistical significance was set at *P*<.05.

^b^HF: hay fever.

^c^DE: dry eye.

^d^IQR: interquartile range.

^e^N/A: not applicable.
