# Supplementary material for: Using the AllerSearch Smartphone App to Assess the Association Between Dry Eye and Hay Fever: mHealth-Based Cross-Sectional Study
Source: J Med Internet Res. 2023 Sep 12;25:e38481. doi: 10.2196/38481 (PMC10523221; doi:10.2196/38481)
Supplement: Multimedia Appendix 6 [file jmir_v25i1e38481_app6.docx]

**Multimedia Appendix 6.** Symptoms and QoL of Dry eye and hay fever in each stratified cluster.

|  | Cluster 1  (n=807) | Cluster 2  (n=864) | Cluster 3  (n=1250) | Cluster 4  (n=923) | Cluster 5  (n= 536) | Cluster 6  (n=1002) | Cluster 7  (n=415) | Cluster 8  (n=682) | Cluster 9  (n=842) | Cluster 10  (n=620) | Cluster 11  (n=736) | Cluster 12  (n=1126) | Cluster 13  (n=531) | Cluster 14  (n=950) |  |
| --- | --- | --- | --- | --- | --- | --- | --- | --- | --- | --- | --- | --- | --- | --- | --- |
| **Scores, median (IQR^a^)** | | | | | | | | | | | | | | | |
| Total NSS^b^, 0-20 | 7 (5-8) | 6 (5-7) | 4 (3-6) | 2 (1-3) | 0 (0-1) | 2 (1-4) | 1 (0-3) | 0 (0-0) | 8 (7-10) | 0 (0-1) | 2 (1-3) | 2 (2-3) | 0 (0-1) | 1 (0-2) |  |
| Total NNSS^c^, 0-16 | 6 (5-8) | 2 (1-4) | 4 (2-5) | 2 (1-3) | 0 (0-1) | 2 (1-3) | 1 (0-2) | 0 (0-0) | 6 (4-8) | 0 (0-1) | 0 (0-1) | 1 (1-3) | 0 (0-1) | 0 (0-1) |  |
| TSS^d^, 0-36 | 9 (7-11) | 7 (6-9) | 6 (4-7) | 3 (2-4) | 0 (0-1) | 3 (1-4) | 2 (0-4) | 0 (0-0) | 11 (9-12) | 0 (0-1) | 2 (2-3) | 3 (2-4) | 0 (0-1) | 1 (0-2) |  |
| J-OSDI^e^ total score, 0-100 | 37.5  (31.3-47.9) | 4.2  (2.1-8.3) | 18.8  (14.6-25) | 6.3  (2.1-10.4) | 4.2  (2.1-6.3) | 37.5  (29.2-47.9) | 20.5  (14.6-27.1) | 0  (0-2.1) | 12.5  (8.3-18.8) | 10.4  (8.3-12.5) | 2.1  (0-4.2) | 13.6  (10.4-18.2) | 10.4  (8.3-12.5) | 22.9  (18.8-27.1) |  |
| QoL^f^ total score, 0-68 | 28 (13-43) | 12 (2-25) | 14 (4-26) | 5 (0-14) | 0 (0-4) | 8 (0-21) | 3 (0-13) | 0 (0-1) | 25 (11-39) | 0 (0-5) | 2 (0-7) | 6 (0-15) | 0 (0-4) | 1 (0-9) |  |

^a^IQR: interquartile range.

^b^NSS: nasal symptom score.

^c^NNSS: nonnasal symptom score.

^d^TSS: total symptom score.

^e^J-OSDI: Japanese version of the Ocular Surface Disease Index

^f^QoL: quality of life.
